# Translation Enhancing ACA Motifs and Their Silencing by a Bacterial Small Regulatory RNA

**DOI:** 10.1371/journal.pgen.1004026

**Published:** 2014-01-02

**Authors:** Qi Yang, Nara Figueroa-Bossi, Lionello Bossi

**Affiliations:** Centre de Génétique Moléculaire du CNRS, Associé à l'Université Paris-Sud, Gif-sur-Yvette, France; Universidad de Sevilla, Spain

## Abstract

GcvB is an archetypal multi-target small RNA regulator of genes involved in amino acid uptake or metabolism in enteric bacteria. Included in the GcvB regulon is the *yifK* locus, encoding a conserved putative amino acid transporter. GcvB inhibits *yifK* mRNA translation by pairing with a sequence immediately upstream from the Shine-Dalgarno motif. Surprisingly, we found that some target sequence mutations that disrupt pairing, and thus were expected to relieve repression, actually lower *yifK* expression and cause it not to respond to GcvB variants carrying the corresponding compensatory changes. Work prompted by these observations revealed that the GcvB target sequence in *yifK* mRNA includes elements that stimulate translation initiation. Replacing each base of an ACA trinucleotide near the center of the target sequence, by any other base, caused *yifK* expression to decrease. Effects were additive, with some triple replacements causing up to a 90% reduction. The enhancer activity did not require the ACA motif to be strictly positioned relative to the Shine-Dalgarno sequence, nor did it depend on a particular spacing between the latter and the initiating AUG. The *dppA* mRNA, another GcvB target, contains four ACA motifs at the target site. Quite strikingly, replacement of all four ACAs by random trinucleotide sequences yielded variants showing over 100-fold reduction in expression, virtually inactivating the gene. Altogether, these data identify the ACA motif as a translation-enhancing module and show that GcvB's ability to antagonize the enhancer function in target mRNAs is quintessential to the regulatory effectiveness of this sRNA.

## Author Summary

The majority of small RNA (sRNA) regulators in bacteria act by inhibiting translation initiation in target messenger RNAs. The study of this regulatory mechanism not only allows a better understanding of sRNA function but it can also provide new insight into aspects of the translation initiation process that remain incompletely characterized. This was the case in the work described here. Analyzing the mechanism by which GcvB, a multi-target sRNA, downregulates a putative amino acid transporter in *Salmonella*, we discovered that the sequence base-pairing with GcvB in the target mRNA functions as a translational enhancer. Replacing an ACA motif near the center of the sequence with unrelated trinucleotide sequences leads to a decrease in translational initiation efficiency that can be as severe as more than 90%. Interestingly, some of these replacements concomitantly render the mRNA insensitive to GcvB variants carrying the appropriate compensatory changes, suggesting that targeting the enhancer element is paramount for GcvB regulatory effectiveness. Overall the data presented in the paper unveil the role of the ACA motif in the translation initiation process and lay the grounds for further analysis of the mechanism involved.

## Introduction

A relevant chapter in the expanding field of RNA-mediated gene regulation is devoted to the activities of multi-target trans-encoded small RNAs in bacteria. Acting in concert with chaperon protein Hfq, these RNA regulators function by base-pairing with short, often imperfectly complementary sequences in the 5′ untranslated regions (UTR) of target messenger RNAs. They can affect translation and turnover of several mRNAs simultaneously thus reprogramming gene expression of whole gene families in a coordinate manner in response to environmental cues (reviewed in [1]–[3]). Archetypal examples of this class of regulators are the RyhB small RNA (sRNA) which represses expression of mRNA for dispensable iron-sequestering proteins when iron is limiting [4]–[8]; RybB, which downregulates several outer membrane protein mRNAs under envelope stress conditions [9]–[13], Spot 42, which amplifies the regulatory range of catabolite repression by targeting several mRNAs involved in sugar uptake and consumption [14] and GcvB, which downregulates dozens of different mRNAs involved in amino acid uptake or metabolism in *E. coli* and *Salmonella* [15]–[18].

GcvB, a 200 nucleotide-long sRNA, was identified serendipitously during a study of *gcvA*, the gene for the main transcriptional regulator of the glycine cleavage operon *gcvTHP* [18]. The latter encodes the enzymes of the glycine cleavage system, the pathway generating one-carbon units from the oxidative cleavage of glycine [19]. The *gcvB* gene is located immediately adjacent to *gcvA* in the opposite orientation with its promoter partially overlapping the *gcvA* promoter. In the presence of excess glycine, the GcvA protein activates transcription of the *gcvTHP* operon as well as of *gcvB* [18]. Initial characterization of GcvB showed this sRNA to downregulate the synthesis of DppA and OppA proteins, main components of dipeptide- and oligopeptide-transport systems, respectively [16], [18]. Since then, the number of genes found to be regulated by GcvB has increased exponentially. A recent transcriptomic study in *Salmonella enterica* set this number to more than 40, making the GcvB regulon the largest of its kind [17]. The vast majority of these loci are linked directly or indirectly to amino acid metabolism and are negatively controlled by GcvB. Typically, regulation is exerted during exponential growth in nutrient rich environments and possibly aimed at coordinating the expression of interconnected metabolic pathways [16], [17]; however, its precise role remains incompletely understood.

GcvB uses a specific sequence region to pair with most, although not all [20] of its mRNA targets. This pairing domain – named the R1 region [16] – is characterized by its high degree of sequence conservation, the lack of secondary structure and a typical GU-rich sequence bias. Hence, most sequences targeted by GcvB include CA-rich repeats. They are typically found inside, or immediately adjacent to, the ribosome binding sites (RBS) of target mRNAs. In one of these targets - the *gltI* mRNA for a glutamate-aspartate transport protein – the CA-rich element is located 45 nucleotides (nt) upstream from the translation initiation codon. Removal of this sequence (as part of a 27 nt deletion), besides causing the loss GcvB regulation, affected *gltI* translation, suggesting that the CA-rich element acts as a translational enhancer. Consistent with this interpretation, crafting the 27 nt segment at the corresponding position of an unrelated mRNA conferred simultaneously GcvB control and increased translational efficiency [16].

Some years ago, our laboratory performed a *lac*-based genetic screen aimed at identifying genes controlled by trans-encoded small RNAs in *Salmonella*. A random library of *lacZ* fusions to chromosomal genes was generated using a phage Mu-derived transposon (MudK) and screened for isolates whose LacZ levels changed (either increased or decreased) upon inactivating Hfq [21]. Among the candidates that were found, two independent isolates upregulated in the *hfq* mutant background, carried the *lacZ* insert translationally fused to the *yifK* gene [21]. Presumptive identification of this gene as an amino acid transporter suggested that *yifK* might be a GcvB target. We thus proceeded to test this hypothesis and characterize *yifK* regulation. While this work was underway, Sharma and coworkers identified *yifK* mRNA as a member of the *gcvB* regulon by microarray analysis; however, these authors could not confirm direct regulation by GcvB due to low reporter fluorescence of the *yifK*-*gfp* fusion used in the study [17]. Since this study also identified global regulator Lrp as a GcvB target [17], the possibility remained that the GcvB effects on *yifK* expression might be indirect.

Here we present *in vivo* and *in vitro* evidence that GcvB downregulates *yifK* directly by pairing with a sequence immediately preceding the Shine-Dalgarno (SD) motif in *yifK* mRNA. A surprising observation in the course of this study was that some target sequence mutations that disrupted pairing did not cause *yifK* expression to increase – as expected for the relief of GcvB repression – but had the opposite effect. The drop in expression was not suppressed by deleting *gcvB* nor was it accentuated in a GcvB mutant carrying the appropriate compensatory changes. Closer analysis revealed that the GcvB target sequence includes elements that stimulate *yifK* mRNA translation. In the absence of such elements, the role of GcvB pairing in regulation becomes marginal.

## Results

### Genetic identification of a GcvB-regulated locus

**Figure 1:**
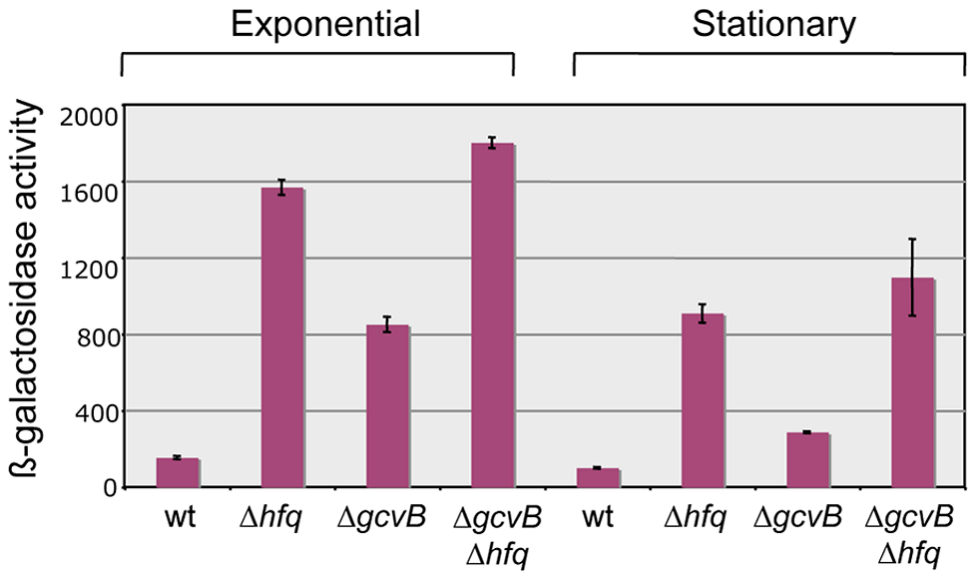
Effect of *hfq* and *gcvB* deletions on the expression of a *yifK*-*lacZ* fusion. Deleting *gcvB* causes *yifK* expression to increase approximately five-fold in exponential cultures (OD600≈0.4) and less than three-fold in stationary overnight cultures (OD600≈2.0). A greater increase is observed in the *hfq* deletion mutant, suggesting the involvement of a separate Hfq-dependent step in *yifK* regulation. Strains used were MA8020 (wt), MA8021 (Δ*hfq*), MA10377 (Δ*gcvB*) and MA10403 (Δ*hfq* Δ*gcvB*). All strains carry the *yifK*::MudK lac fusion. Their full genotypes are listed in Table S1.

Our original screen for Hfq-regulated genes yielded two isolates carrying the MudK (*lac*) transposon in the *yifK* gene; one predicted to produce a LacZ protein fusion to the 48^th^ amino acid (aa) of the 461 aa YifK (*yifK87*::MudK); the other with LacZ inserted after the 95^th^ aa of YifK (*yifK88*::MudK) [21]. Preliminary tests showed both fusions to be regulated in a closely similar manner; however, *yifK87*::MudK produced significantly higher ß-galactosidase activity and was chosen for the present study. A survey of protein sequence databases showed YifK to be a highly conserved protein with the characteristic signature of amino acid transporters. The known role of GcvB in the regulation of some members of this family made this small RNA the likeliest candidate to control *yifK* expression. This was confirmed by deleting the *gcvB* gene and testing the effects of the deletion on the expression of the *yifK87*::MudK fusion (hereafter referred to as *yifK*-*lacZY*). As shown in Figure 1, the *gcvB* deletion causes a nearly 5-fold increase of ß-galactosidase activity in exponentially growing cells, while effects decline in stationary phase. Somewhat surprisingly, LacZ levels in the *gcvB*-deleted strain are not as high as the levels measured in a strain deleted for *hfq* (Figure 1). This might reflect the existence of one or more additional sRNA(s) participating in *yifK* repression. Alternatively, Hfq could repress *yifK* directly [22]. The data in Figure 1 show that loss of Hfq is epistatic to the *gcvB* deletion.

### Mutations affecting *yifK* expression

**Figure 2:**
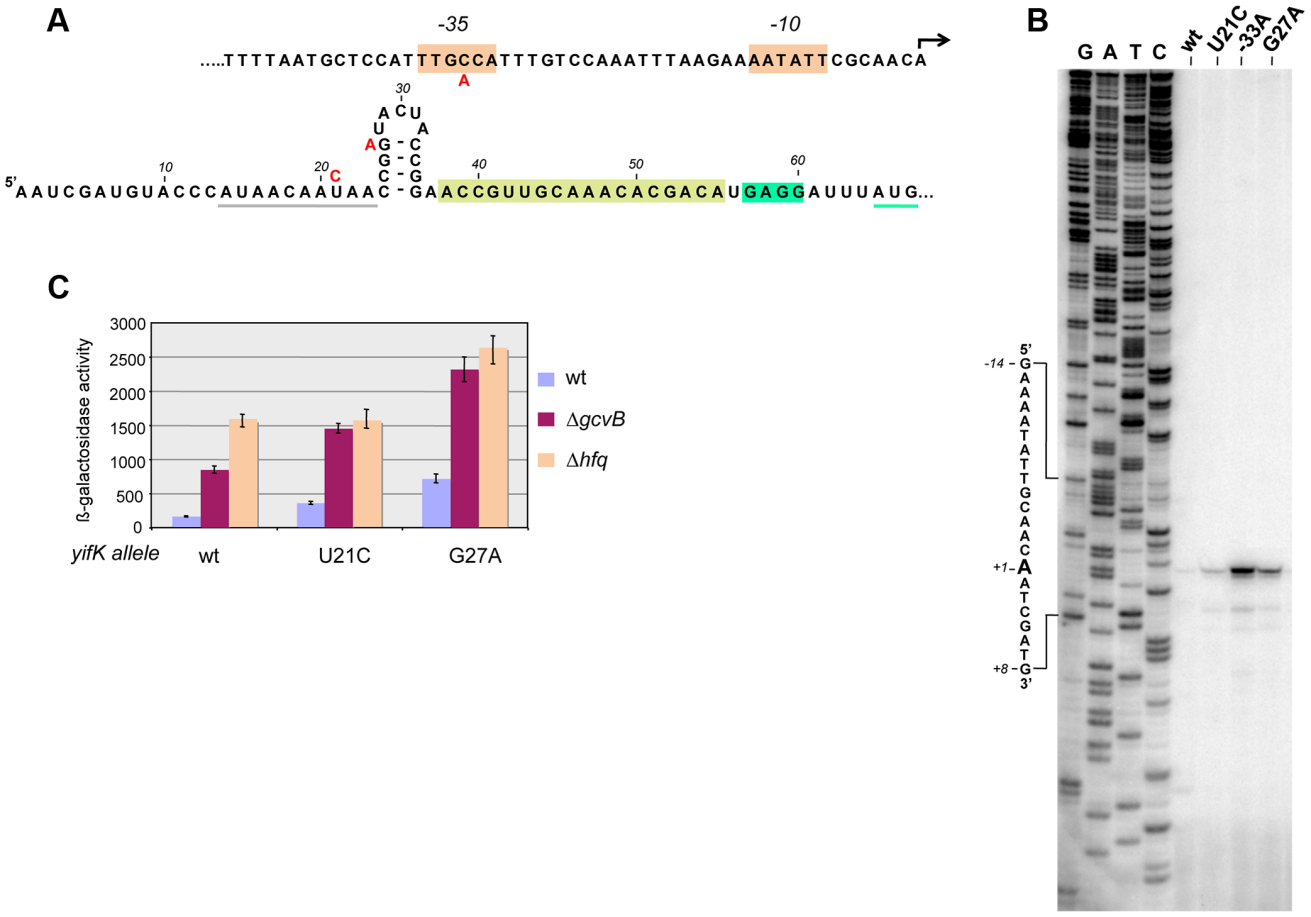
Characterization and mutational analysis of the leader region of the *yifK* gene. The promoter-proximal region of the *yifK* gene was randomly mutagenized by error-prone PCR using primers ppF45 and ppF47 and chromosomal DNA from a strain carrying a *ca*t cassette 78 bp upstream from the *yifK* promoter (in opposite orientation; strain MA11780) as template. The mutagenized fragment was introduced into strain MA10280 (*yifK-lacZY*/pKD46) and recombinants were selected as described in the text. Three mutants expressing higher ß-galactosidase activity were identified: one carrying a *yifK* promoter change that causes the -35 box of *yifK* to match the consensus sequence (TTGACA) (A, top); the other two isolates carrying mutations in the 5′UTR (U to C at +21 and G to A at +27) (A, bottom). Grey and green underlining denote a putative Hfq binding site and the initiating AUG, respectively. Brown overlining denotes the Shine-Dalgarno sequence. A sequence stretch complementary to GcvB is boxed in light green. B. Primer extension of *yifK* mRNA. Total RNA extracted from wild-type and mutant strains was used to map the 5′ end of the *yifK* mRNA by reverse transcription (primer ppF49)(B). This analysis identifies the 5′ end of *yifK* mRNA and shows that all three mutations lead to higher mRNA levels. Measurements of ß-galactosidase activity (C) show that U21C, but not G27A, does not cause any further increase in *yifK-lacZ* expression in the Δ*hfq* background, suggesting that U21C affects Hfq binding to *yifK* mRNA. ß-galactosidase activity was measured in exponentially growing cultures (OD600≈0.4).

Primer extension experiments mapped the 5′ end of *yifK* mRNA to 64 nucleotides upstream from the initiating AUG (Figure 2). This 5′ untranslated region (UTR) includes a 14-nt stretch complementary to the 3′ half of GcvB's R1 region. As an initial step to characterize GcvB involvement in *yifK* regulation, we tested whether point mutations in the *gcvB* gene or in the promoter-proximal portion of *yifK* relieved GcvB-mediated repression. For this, DNA fragments spanning either of these two regions were randomly mutagenized by the polymerase chain reaction (PCR) under error-prone conditions and introduced into the chromosome of a strain harboring the *yifK-lacZY* reporter fusion via lambda *red* recombination. Most of the isolates originating from the *gcvB* mutagenesis carried changes in the *gcvB* promoter or in the promoter of the adjacent *gcvA* gene (Figure S1). Thus, these mutations appeared to lower the levels rather than the activity of GcvB and were not further considered.

Mutagenesis of *yifK* promoter-proximal segment yielded three mutants with elevated *yifK-lacZY* expression. One isolate carried a C:G to A:T change 33 base-pairs upstream from the 5′ end of *yifK* mRNA. The position and the nature of the change (producing a -35 promoter consensus match, TTGACA, Figure 2A), strongly suggest that the mutation increases the strength of the *yifK* promoter. The mutation leads to a sharp rise in the intensity of the primer extension product (lane “-33A” in Figure 2B) and a more than 10-fold increase in ß-galactosidase activity (data not shown). These findings confirmed that the 5′ end identified by primer extension corresponds to *yifK* transcription initiation site. The remaining two mutations affect residues within the 5′ UTR (Figure 2A). One allele, resulting in a U to C change at position +21, falls within a AU-rich segment (AUAACAAUAA) that might constitute a site for Hfq binding [3], [23]. Consistent with this interpretation, the mutation has no effect in Δ*hfq* background (Figure 2C). Finally the third allele (G to A at +27) affects the CG-rich stem of a presumptive secondary structure immediately adjacent to the AU-rich segment. The change causes a generalized increase of *yifK-lac*ZY expression by an unidentified mechanism.

### GcvB activity stimulates RNase E-dependent *yifK* mRNA decay

**Figure 3:**
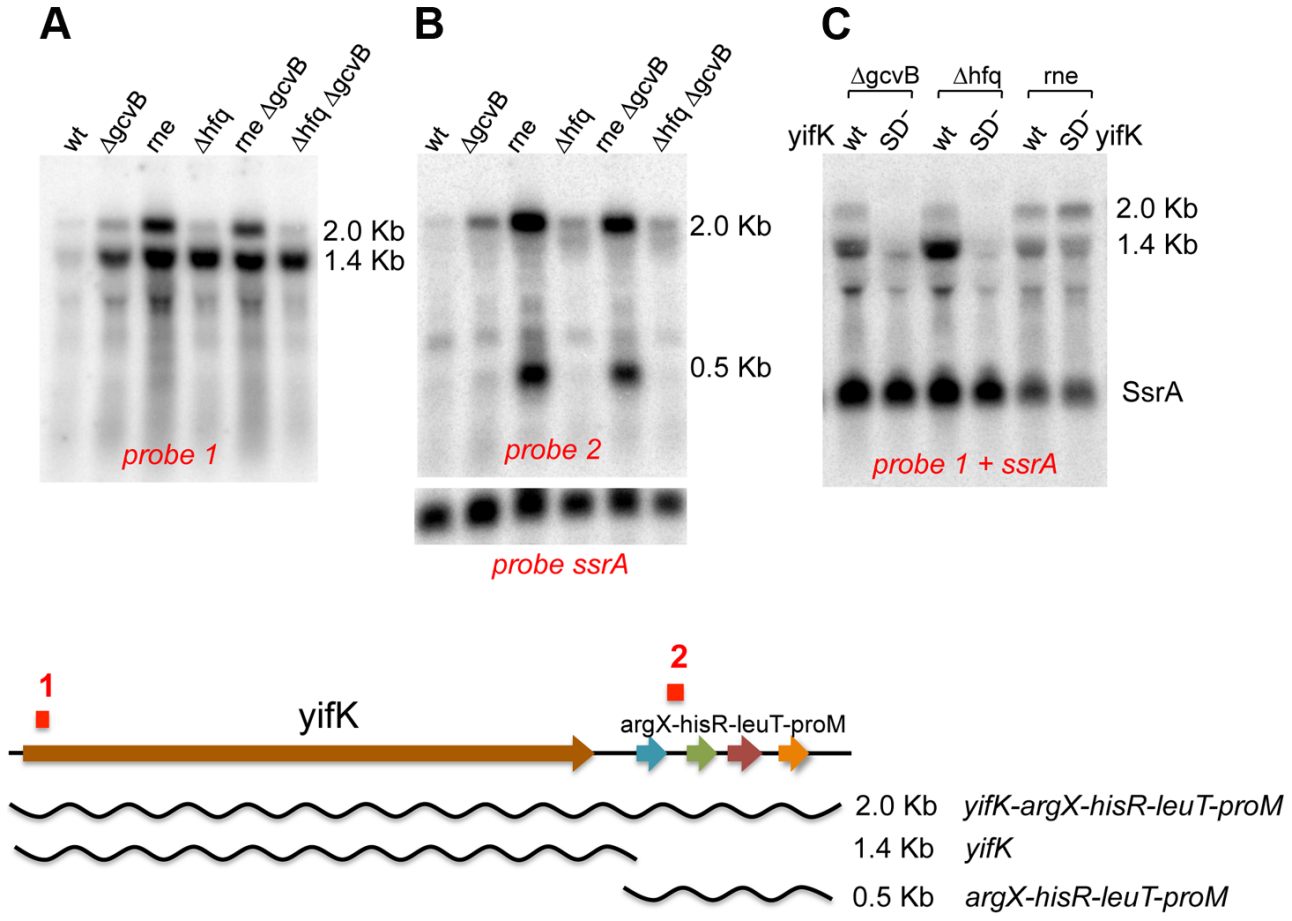
Northern blot analysis of *yifK* transcription. All strains used as source of RNA carried -33 the promoter “up” mutation. The RNAse E mutant carried the temperature sensitive (ts) *rne-3071* allele [28]. In experiments involving this strain, bacteria were grown at 30°C and shifted to 43°C 15 min prior to RNA extraction. RNA was separated on a 1% agarose-formaldehyde gel and probed with ^32^P-labeled DNA oligonucleotides complementary to a sequence near the 5′ end of *yifK* mRNA (ppF16; probe 1 above) or to a sequence in the *argX-hisR* intercistronic region (ppH27, probe 2). The blot in A and B was initially probed with ppF16 (A), then stripped and re-probed with ppH27 (B). Probing for the SsrA RNA (pp813) served as loading control. The blot in C was probed simultaneously with ppF16 and pp813. “SD^−^” denotes a G to C change in the Shine-Dalgarno sequence (+59), which causes an about 10-fold reduction in *yifK* expression (construct n. 2 in Figure 9).

Northern blot analysis was used to assess the effects of GcvB regulation on *yifK* mRNA levels. This study critically benefited from the availability of the -33 promoter mutant (see above), *yifK* mRNA being otherwise undetectable when expressed from the wild-type promoter (data not shown). The analysis identified two *yifK* mRNA species, a 1.4 kilobase (Kb) mRNA covering just the *yifK* coding portion and a longer, 2.0 Kb RNA extending into the adjacent *argX-hisR-leuT-proM* tRNA operon. As shown in Figure 3A, both RNAs accumulate upon RNase E inactivation, whereas only the shorter species accumulates in cells lacking GcvB or Hfq. This suggested that derepression of *yifK* translation in Δ*gcvB* or Δ*hfq* cells protects the 1.4 Kb RNA against RNase E cleavage. To confirm this interpretation, the analysis was repeated with strains that, besides the promoter “up” mutation, carried a mutation in the Shine-Dalgarno sequence (G to Cat position +59; described in more detail below). As shown in Figure 3C, the SD mutation causes the intensity of 1.4 Kb band to sharply decrease in the Δ*gcvB* or Δ*hfq* strains but not in the *rne* ts mutant, consistent with the idea that reduced translation renders *yifK* mRNA susceptible to RNAse E degradation.

Absence of any obvious transcription termination signals in the intercistronic region between *yifK* and the tRNA operon suggests that the 1.4 Kb RNA originates from processing of the longer form. Likely, under normal conditions (*i.e.*, wt *yifK* promoter) *yifK* transcription contributes only to a small fraction of the four tRNAs, as most the tRNA operon transcription results from a strong promoter located immediately upstream from the *argX* gene [24]. The approximately 500 nt RNA accumulates in the RNase E mutant (Figure 3B). Previous work in *E.coli*, showed that this tRNA precursor is processed by the concerted actions of RNase E and RNase P in a pathway that, intriguingly, also sees the participation of Hfq [25].

### *yifK* is repressed by Lrp

**Figure 4:**
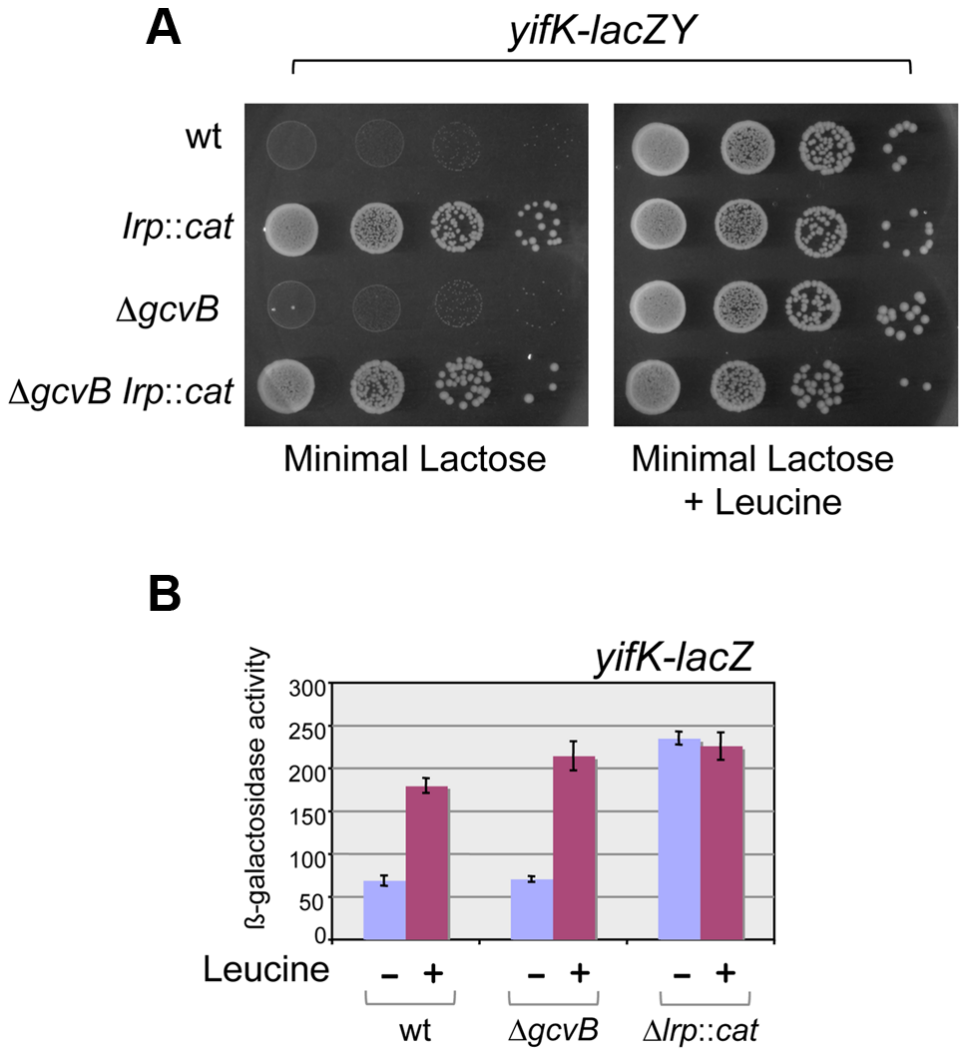
Lrp control of *yifK-lacZY* expression. The *yifK* gene is expressed at very low levels in minimal medium. As a result, strains carrying the *yifK-lacZY* fusion are phenotypically Lac^−^ in this medium, regardless of the *gcvB* allele (A, left panel). Inactivation of Lrp confers a Lac^+^ phenotype (A, left panel). This suggests that low *yifK* expression results from Lrp repression. The Lac^+^ phenotype is also restored upon addition of leucine (0.3 mM) (A, right panel), indicating that leucine relieves Lrp repression. ß-galactosidase measurements (B) confirm that GcvB plays no significant role in *yifK* regulation in minimal medium and provide a quantitative estimate of the Lrp effects. NCE medium [43] supplemented with 0.2% lactose (A) or 0.2% glycerol (B) was used as minimal medium.

Early on in this study, it became apparent that *yifK* expression was exquisitely sensitive to the growth medium and virtually silenced in minimal medium. As a result, a strain with the *yifK-lacZY* fusion is phenotypically Lac^−^ when plated in minimal medium. We exploited this phenotype to positively select for spontaneous Lac^+^ mutants. The selection yielded two classes of mutations, one genetically linked to the *yifK-lacZY* locus, the second mapping elsewhere. All of the linked mutants that were analyzed were found to harbor the -33 C:G to A:T promoter change obtained previously (see above). The unlinked mutations mapped in a chromosomal interval encompassing the gene for leucine response regulator, Lrp. Prompted by this observation, we introduced an *lrp* insertion mutation into the *yifK-lacZY*-containing strain. The resulting strain acquired a Lac^+^ phenotype (Figure 4A), indicating that *yifK* silencing in minimal medium results from Lrp repression. Addition of leucine efficiently relieves repression (Figure 4). The data in Figure 4 also show that GcvB does not contribute to *yifK* repression to any significant extent in minimal medium. This is not surprising as GcvB is transcribed at very low level under these conditions [16] and inactivating Lrp does not reverse this pattern (Figure S2). The data in Figure S2 differ from those of Modi et al [26] who reported an approximate 30-fold increase in GcvB levels in an *lrp* deletion mutant of in *E.coli*. This discrepancy might reflect differences in the organisms used or in media composition.

### GcvB inhibits *yifK* translation by targeting an enhancer element

**Figure 5:**
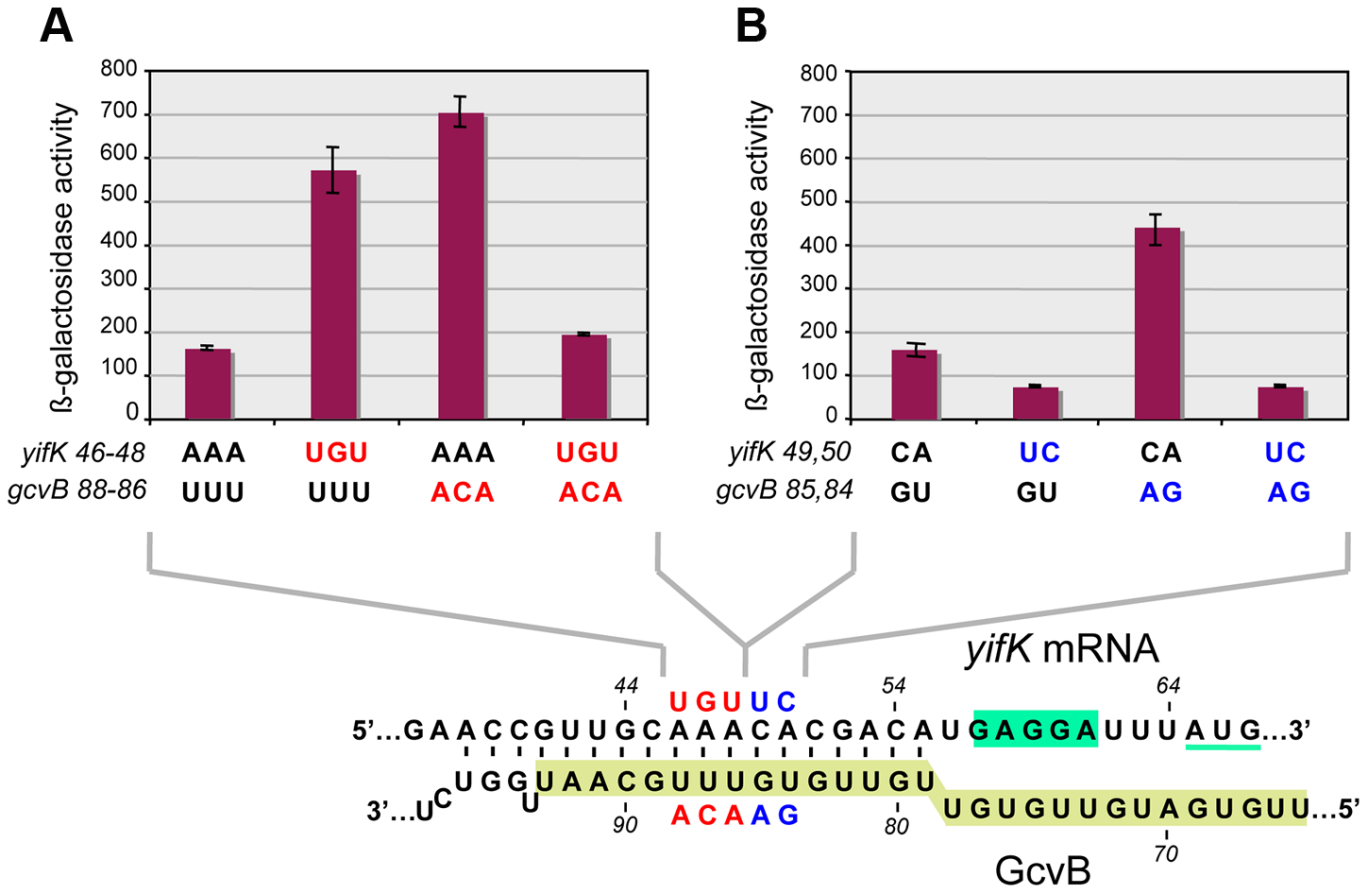
Differential effects of target sequence mutations on *yifK-lacZ* expression and on the response to compensatory changes in GcvB. Adjacent portions of the sequence presumed to base-pair with GcvB in *yifK* mRNA were randomly mutagenized by a “scarless” λ *red* recombineering procedure (see Materials and Methods). Briefly, DNA fragments amplified by “reciprocal priming” with oligonucleotide pairs ppG48/ppG49 (mutagenesis of +46 to +48) and ppH12/ppH13 (mutagenesis of +49, +50) were introduced into strain MA11526 and tetracycline-sensitive recombinants selected one plates supplemented with fusaric acid (12 µg/ml). Two of the mutants obtained were chosen for further study. Compensatory changes in GcvB were obtained by standard recombineering using fragments amplified from the chromosome of strain MA11779 with primer pairs ppG63/ppF18 (mutagenesis of the +86 to +88) and ppH61/ppF18 (mutagenesis of +84, +85). Results above show that changing *yifK* mRNA sequence from positions +46 to +48, or making the opposite change in GcvB, both relieve repression (A). Repression is restored in a strain carrying the two sets of changes combined, showing that base-pairing is required for repression (A). In contrast, replacing the CA doublet at +49, +50 by UC causes a reduction, rather than an increase, of *yifK-lacZ* expression; introduction of the compensatory changes in GcvB does not accentuate this trend (B). Thus, the CA to UC conversion impairs *yifK* expression and renders it insensitive to GcvB repression.

**Figure 6:**
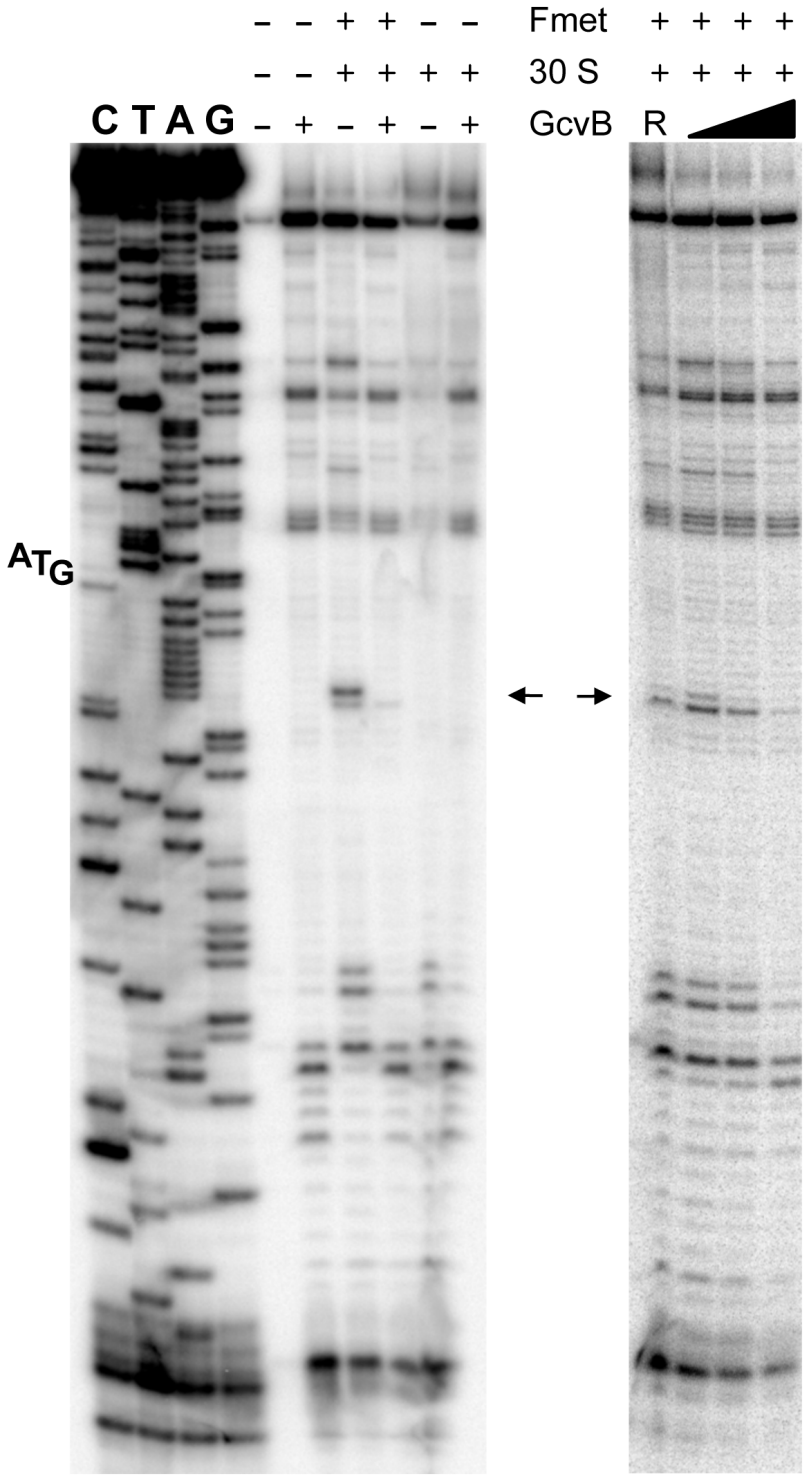
Toeprinting analysis of *yifK* mRNA. 30S ribosomal toeprinting was carried out in the absence or in the presence of GcvB RNA or of RyhB RNA as described in the Materials and Methods. “+” and “−” signs denote the presence of absence of indicated components. The decline and disappearance of the toeprint (shown by arrows) at increasing GcvB concentrations (50 nM, 100 nM and 500 nM), is indicative of interference with the 30S subunit binding to *yifK* mRNA. Failure of RyhB to do so at the concentration of 6.0 µM (lane “R”) confirms the specificity of the effect.

The above approach yielded no mutations affecting the presumptive pairing sequences of GcvB or *yifK*. Reasoning that single base changes might not disrupt regulation enough to be revealed by the MacConkey plate screen, we resorted to introducing multiple changes by site-directed mutagenesis. An initial test involved changing a UGUG quadruplet in the GcvB segment thought to pair with *yifK* mRNA. The alteration caused expression of the *yifK-lacZY* fusion to increase approximately threefold, thus corroborating the postulated role of this sequence in *yifK* repression. Unexpectedly, however, when the ACAC sequence at the corresponding position in *yifK* mRNA was changed, *yifK-lacZ* expression did not increase but actually declined (data not shown). Trying to clarify this observation, portions of the region of interest were mutagenized separately. As shown in Figure 5A, converting the AAA sequence in the middle of the target sequence to UGU, or making the opposite change (UGU to AAA) in GcvB, similarly relieves *yifK-lacZY* repression. Repression is restored upon combining the compensatory alleles. Thus, this portion of the target sequence behaves as expected, and the behavior of the compensatory mutant strongly suggests that GcvB represses *yifK* through a base-pair interaction. *In vitro* toeprint experiments, showing that GcvB inhibits the binding of ribosomal 30S subunit to *yifK* translation initiation site, specifically and in a dose-dependent manner (Figure 6), provided independent support to this conclusion.

Again, however, changing the CA doublet on the 3′ side of the AAA sequence to UC produced an unusual pattern: like in the quadruplet mutant above, *yifK-lacZY* expression decreased rather than increase, becoming insensitive to a GcvB variant carrying the compensatory change (Figure 5B). To verify that the compensatory change did not hamper GcvB's function in an unpredictable way, we took advantage of the fact that the replaced nucleotides do not participate in the pairing with *dppA* [16] and tested the mutant's ability to repress a *dppA-lacZ* translational fusion. This analysis showed both GcvB variants to be as efficient as wild-type in repressing *dppA*, indicating that both alleles remain fully functional (Figure S3).

**Figure 7:**
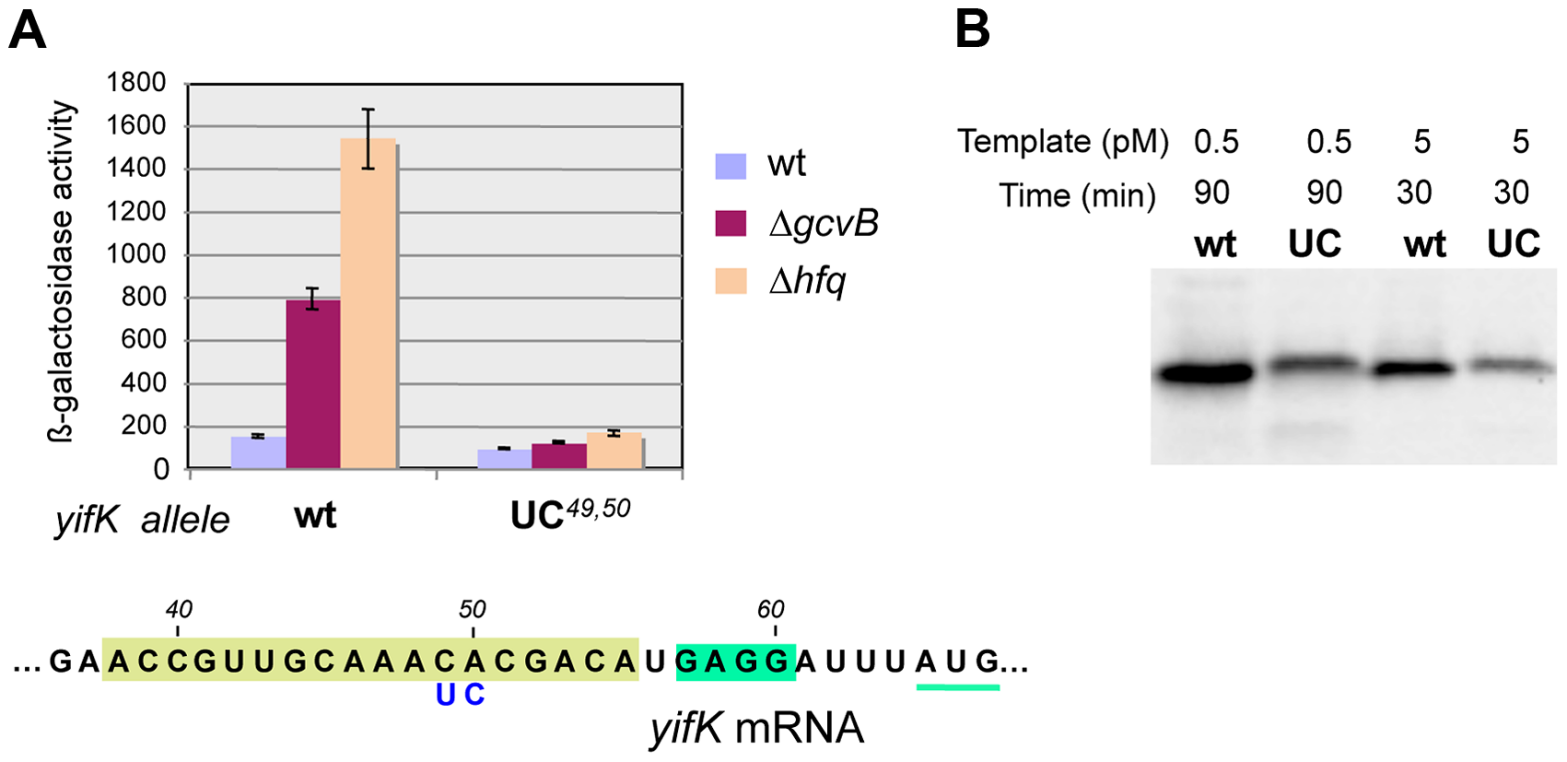
Effect of *yifK* 5′ UTR's changes on mRNA translation. The CA*^49,50^* to UC*^49,50^* change in *yifK* mRNA affects expression levels *in vivo* (A) and mRNA translation *in vitro* (B). *In vitro* translation was performed using the coupled transcription/translation PURExpress kit (see Materials and Methods). DNA templates were from plasmids carrying the entire *yifK* 5′ UTR from wild-type, or from the UC*^49,50^* mutant, fused to the coding sequence of a 3×FLAG epitope-tagged version of the *cat* gene. Fusions were initially obtained as chromosomal constructs using DNA fragments amplified from strain MA7224 with primer pairs ppL50/ppL52 (wt) and ppL51/ppL52 (UC*^49,50^*). Subsequently, the fusions were cloned into plasmid DHFR following amplification (ppM29/ppM30) and double Xba I/Pst I digestion. Transcription/translation reactions were carried out at a template DNA concentration of 0.5 pM for 90 min or 5 pM for 30 min and products analyzed by Western blotting using anti-FLAG monoclonal antibodies [53]. Under both conditions, higher amounts of *cat*-3×FLAG protein were synthesized from the construct with the wild-type *yifK* sequence than from the construct harboring the CA*^49,50^* to UC*^49,50^* change.

Besides being insensitive to the compensatory GcvB allele, the *yifK* CA*^49,50^* to UC*^49,50^* mutant fails to respond to *gcvB* or *hfq* deletions (Figure 7A). We interpreted these findings to suggest that the CA to UC conversion lowers translation efficiency and under such conditions, GcvB action is no longer rate-limiting for *yifK* expression. The effects of the mutation on *yifK* translation were examined *in vitro* using a reconstituted system. Results in Figure 7B showed an epitope-tagged Cat protein to accumulate at significantly greater levels when made from a gene fusion to the wt *yifK* 5′ UTR than from an equivalent construct carrying the CA to UC change. These data confirmed that the CA*^49,50^* doublet stimulates translation and suggested that GcvB effectiveness in regulation reflects the targeting of an activating element.

### Anatomy of a translational enhancer

**Figure 8:**
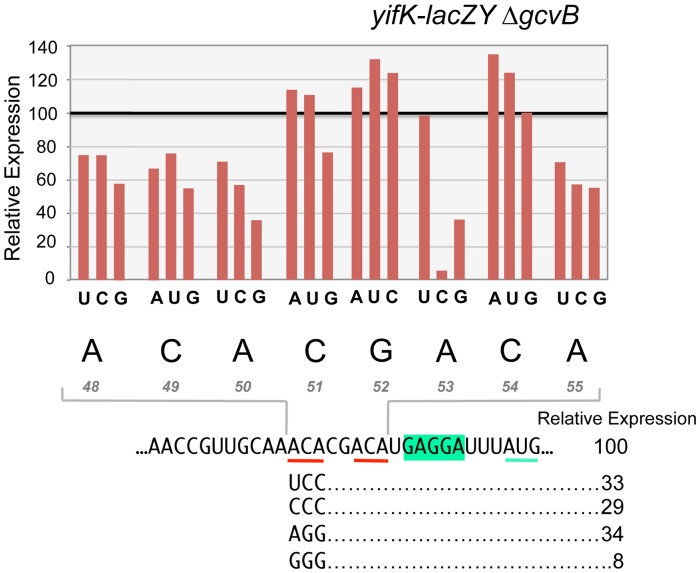
Mutational analysis of the enhancer element. Each base of an 8-nt sequence spanning positions +48 to +55 in *yifK* mRNA was randomized and changes were introduced in the chromosome of strain MA11594 (*yifK-lacZY* Δ*gcvB*) as described in the legend to Figure 5 (primers used to generate the set of mutagenized fragments are listed in Table S2). Mutants obtained were screened by PCR (primers ppF45/ppF62) and DNA sequencing. All possible replacements (a total of 24 mutants) were identified. These strains were assayed for ß-galactosidase activity. Typically, duplicate or triplicate ß-galactosidase measurements were carried out in parallel for all variants of any given position and the wild-type strain, whose value was set to 100. Standard deviations were less than 5% of the mean in all cases. Representative examples of triple substitutions in the upstream ACA are also shown.

To characterize the enhancer element, an 8-nt segment preceding the SD sequence was modified by systematically changing individual residues to each of the three alternative bases. Effects on expression of the *yifK-lacZ* fusion were measured in a strain deleted for the *gcvB* gene. As shown in Figure 8, any change in the ACA sequence at positions +48 to +50 lowers *yifK-lacZY* expression. Variations range between 23% and 62%, with G residues exerting the most adverse effects at any position. Significantly, the effects appear to be additive since a separate experiment in which all three bases in the ACA sequence were randomized yielded alleles undergoing as much as 92% reduction in *yifK-lacZY* expression (bottom portion of Figure 8).

Alteration of a second ACA motif between +53 and +55 produced a somewhat different pattern. Changes in the central C were either neutral or stimulatory; in contrast, having a C at the first position was highly deleterious resulting in nearly 95% reduction of ß-galactosidase activity (Figure 8). Altogether, these data suggested that ACA motifs enhance the efficiency of *yifK* translation. Conservation of these motifs in distant members of the *Enterobacteriaceae* family (Figure S4) is consistent with their functional importance.

**Figure 9:**
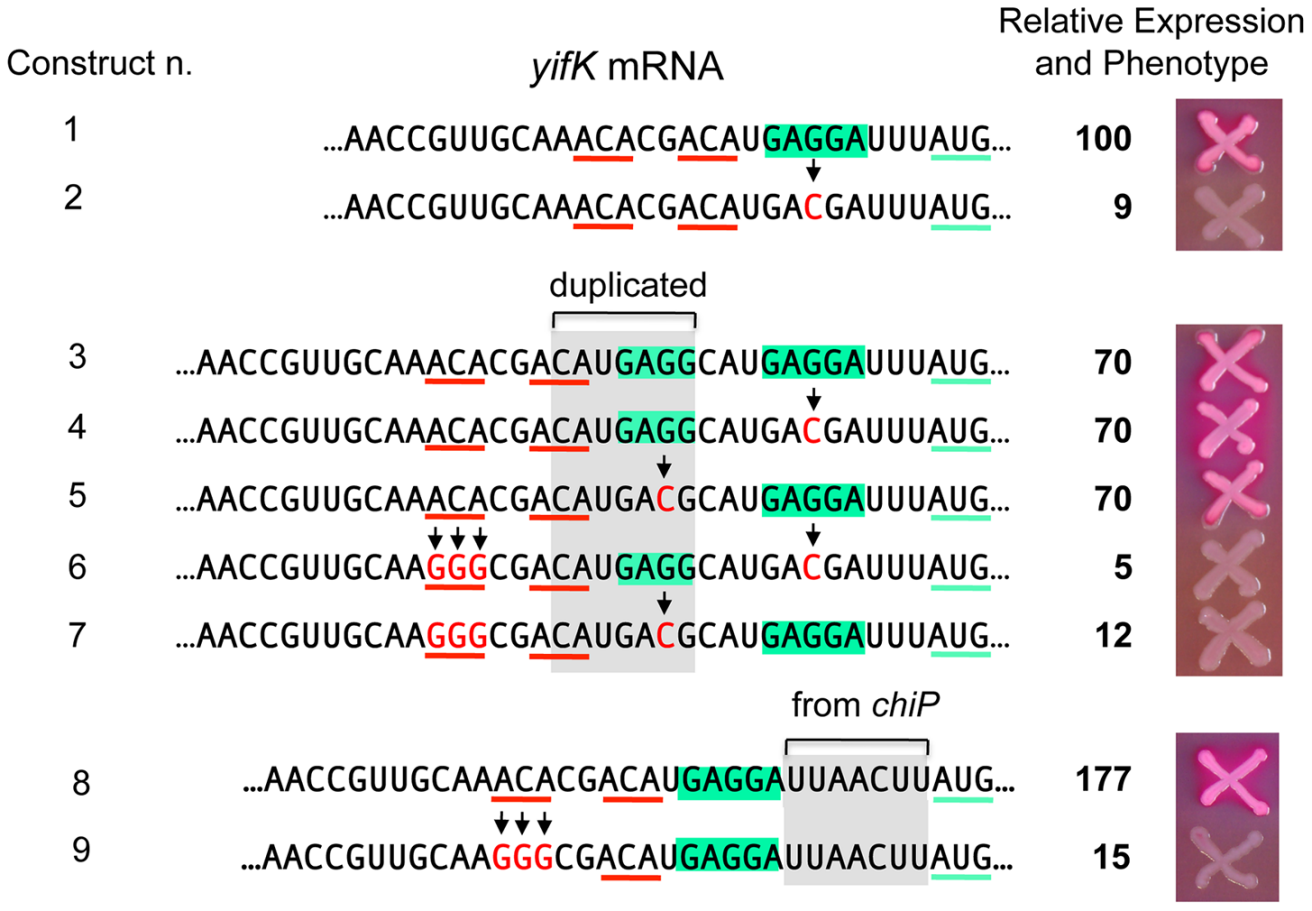
Increasing the distance between the enhancer and the translation initiation region. Constructs were made as described in the legend to Figure 5 using strain MA11594 (*yifK*-*lacZY* Δ*gcvB*) as recipient and fragments amplified by reciprocal priming of oligonucleotides described in Table S2. A 7-nt segment (boxed in grey), duplicating the SD sequence (boxed in green), was inserted between the ACA triplets (underlined in red) and the SD sequence (construct n. 3). A G to C change was then introduced in either copy of the SD (constructs n. 4 and 5; construct n. 2 shows the effect of this change in a strain with a single SD). The upstream ACA was converted to GGG in the constructs carrying either SD sequence mutated (constructs n. 6 and 7). The same procedure was used to replace the SD-AUG interval of *yifK* with the corresponding segment from the *chiP* gene (constructs n. 8 and 9). ß-galactosidase activity was measured as described in the legend to Figure 8. The activity of the wild-type strain (construct n. 1) was set to 100. Standard deviations were less than 5% of the mean in all cases. The data (see also strains' phenotypes on MacConkey-lactose plates) show that the upstream ACA maintains its enhancer effect when placed further upstream from the initiation region, independent of the spacing between the SD sequence and the starting AUG.

A peculiarity of the *yifK* translation signal is the unusually short distance (four nucleotides) between the most conserved base of the SD motif [27] and the initiating AUG. We thus envisaged that the role of the enhancer could be to somehow compensate for such suboptimal arrangement. To test this possibility, we generated a 7-nt tandem direct duplication of the SD region and then inactivated either copy of the SD by changing the GAGGA motif to GACGA (Figure 9). Thus, the resulting constructs have the functional SD sequence positioned either 4 or 11 nt from the AUG. As shown in Figure 9, these two variants (n. 4 and n. 5) express ß-galactosidase levels that are similar to each other and to the strain in which both SD are functional (n. 1). However, when the upstream ACA motif is replaced by GGG, *yifK-lacZ* expression drops sharply in both constructs (compare n. 4 ton. 6, and n. 5 ton. 7). Similar effects were observed in a separate construct where the segment between SD sequence and the initiating AUG was replaced by the sequence found at the corresponding position in the *chiP* gene [28] where the spacing (9-nt) is optimal (n. 8 and n. 9). While construct n. 8 is tightly repressed by GcvB, its variant lacking the upstream ACA (n. 9) shows a weak response to this sRNA (Figure S5). In conclusion, these results indicate that the enhancer activity does not require strict positioning of ACA relative to the initiation site, nor it depends on the spacing between the SD and the initiation codon. Loss of the enhancer function causes y*ifK* expression to be less sensitive to repression by GcvB.

### Essential role of ACA motifs in *dppA* expression

**Figure 10:**
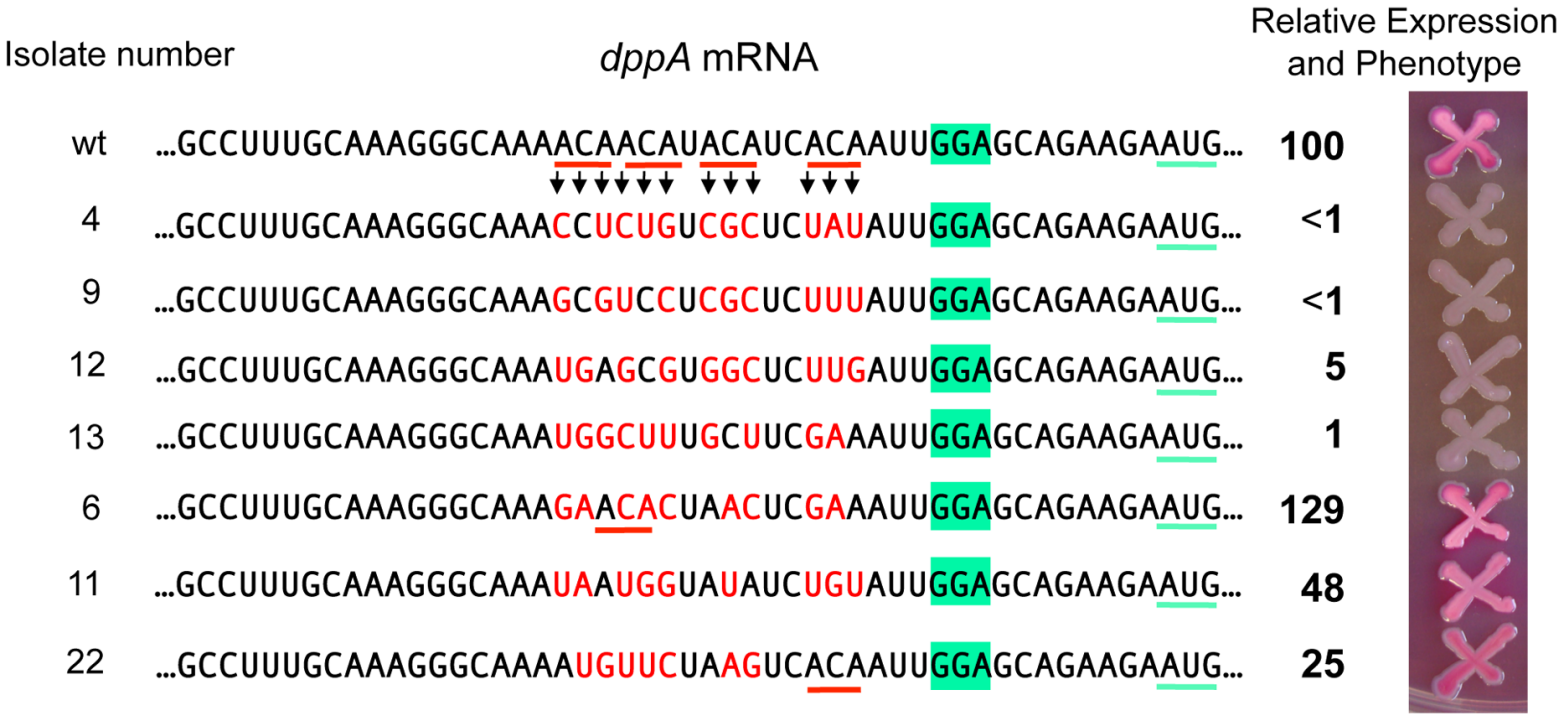
Randomizing ACA motifs in the ribosome binding site of *dppA* gene. A *dppA-lacZ* translational fusion was constructed by converting a KanR insertion derived from plasmid pKD13 [47] to a *lac* fusion using plasmid pCE40 [56]. A *tetAR* insertion deleting the 15 bp ACA-encoding segment was constructed using a fragment amplified with primer pair ppN82/ppN83 (Tables S2 and S3). Next, the *tetAR* insert was replaced with a PCR-amplified fragment (reciprocal priming of ppN85/ppN86) containing a randomized sequence in the ACA-encoding portion. Tetracycline-sensitive recombinants were selected as described ([52], see Materials and Methods) and subsequently screened on MacConkey-lactose indicator plates. A number of isolates were characterized by DNA sequence analysis and ß-galactosidase assays. The activity of the wild-type strain was set to 100.

To assess the generality of the ACA effects, we turned to the *dppA* gene, a major GcvB target [16], [18]. The target sequence of GcvB in *dppA* mRNA includes four ACA motifs clustered within a 15 nt segment near the SD sequence (Figure 10). As an initial test, this 15-nt sequence was deleted in a strain carrying a *dppA-lacZY* translational fusion. The resulting mutant showed 96% lower of ß-galactosidase activity than the parental strain (data not shown). In the next experiment, we randomly mutagenized all four ACA repeats (in the same *lacZ* fusion background) and screened the mutants on MacConkey lactose indicator plates. Out of 43 mutants analyzed, 18 formed white colonies and had ß-galactosidase activities ranging between 0.5 and 5% of the wild-type levels. Four representative isolates from this group are shown in Figure 10. They are essentially Lac^−^ mutants. 4 of the initial 43 mutants formed red colonies and expressed significant levels of ß-galactosidase (three shown in Figure 10). Interestingly, in two of these strains, the mutagenic process regenerated an ACA sequence. The remaining 21 isolates had an intermediate phenotype (pink colonies) and were not analyzed. Examination of the mutant sequences by the Mfold algorithm [29] showed a complete lack of correlation between presence/absence of secondary structures (or free energy values) and *lacZ* expression. Hence the most likely conclusion from this analysis is that the variations in *lacZ* expression levels are solely dictated by primary sequence determinants. Although the possibility that decreased expression in some of the mutants could be due to reduced mRNA stability, independent of ribosome binding, cannot be formally ruled out, it seems most likely that the observed differences reflect variations in translation initiation rates. Thus, on one hand, the data in Figure 10 further corroborate the positive role of the ACA motif in translation initiation; on the other hand, they reiterate the notion that an SD motif and a properly spaced AUG are not sufficient to promote initiation if placed in unfavorable sequence contexts [30].

## Discussion

In the present work, we have characterized the regulation of *Salmonella*'s *yifK* locus encoding a putative amino acid transporter highly conserved in *Enterobacteriaceae*. Our analysis showed *yifK* to be negatively controlled at the transcriptional level by the leucine response regulator Lrp, and at the post-transcriptional level by GcvB sRNA. These findings place *yifK* at the intersection of two global regulatory networks devoted to amino acid management [17], [31]. The relative impacts of two systems on *yifK* expression vary as a function of growth conditions, with the Lrp control predominating in leucine-deprived poor media and the GcvB control operating when amino acids are plentiful, possibly in excess. The sole condition where *yifK* appears to escape negative control is leucine-supplemented minimal medium, where Lrp repression is relieved. This response closely parallels that of the oligopeptide permease operon, *oppABCDF* [31] whose transcript is also a target of GcvB repression [16], [18]. Likely, the overlap of Lrp and GcvB networks reflects the link between amino acid metabolism and one-carbon units production; however, the precise physiological role and the implications of the above responses remain incompletely understood.

Genetic analysis of GcvB:*yifK* mRNA interactions revealed that the GcvB target sequence in *yifK* mRNA contains an enhancer element. Intriguingly, mutations that disrupt the enhancer - and lower *yifK* expression as a result - render *yifK* expression totally insensitive to GcvB repression. This suggests that the effectiveness of GcvB regulation is dependent on the enhancer function and that when this component is removed, GcvB-mediated repression no longer constitutes a rate-limiting step in *yifK* expression. Sharma and coworkers (2007) previously showed that GcvB's target sequence in the *gltI* gene of *Salmonella* acts as transferable translation enhancer (see Introduction). Unlike in our study, the effects of GcvB as a translational repressor were much greater than the effects of removing the enhancer, leading the authors to conclude that GcvB did not simply block the enhancer effect [16]. It seems possible that the plasmid-borne nature of the *gcvB* gene in the study by Sharma and coworkers made the GcvB repression tighter than when the sRNA is expressed from the chromosome. Alternatively, the contribution of the enhancer to *gltI* expression might be less important than in *yifK* expression. The *gltI* enhancer, located 45 nt upstream from the initiation codon, was characterized as part of a 27 nt segment and not analyzed in any further detail [16]. Here we found that nucleotide replacement in either of two ACA triplets within GcvB target site in *yifK* can result in more than 90% reduction in *yifK* expression. Although our data do not allow defining the contours of the enhancer element, they unequivocally identify the ACA motif as a determinant of its activity. We also found that the enhancer activity is maintained following a 7 nt shift in the position of the initiation site, suggesting the absence of strict spatial requirements for the functioning of the element. This is consistent with the data from the *gltI* system and with a report showing CA repeats to stimulate translation even when placed downstream from the start codon [32].

Translation initiation efficiencies have been known to vary greatly as a function of the sequence context of the initiation region [30], [33]. Computational analysis of sequences surrounding translation initiation sites of *E.coli* genes showed that the spacing between the SD and the initiation codon affects SD sequence conservation and its pattern. This study did not reveal significant biases outside these main elements [27]; however, conserved patterns occurring at variable positions might have been difficult to identify by the statistical analysis. Indeed, separates lines of evidence point to the role of the ACA motif in translation initiation. The motif is found in other translation enhancer sequences [34], [35] and, as an ACAA repeat, was shown to promote translation initiation in the absence of a SD sequence [36]. ACA is also found in the loops of pseudoknots formed by RNA ligands to ribosomal protein S1, obtained through Systematic Evolution of Ligands by Exponential Enrichment (SELEX) [37] and is part of the SELEX-determined consensus sequence for binding of protein CsrA, a translational regulator [38]. Finally, ACA is the recognition sequence of the MazF endonuclease that inactivates *E.coli* mRNAs by preferentially cleaving near the translation initiation codon [39].

The lack of position requirements for the functioning of the enhancer suggests that its role is to provide an anchor point for the 30 S ribosomal subunit so as to facilitate subsequent recognition of the SD sequence. Some of the evidence reviewed above tentatively identifies protein S1 as the possible candidate for the interaction. In vitro S1-binding studies with some of the mutants constructed in the course of this work should allow testing of this idea. Combined with the mutational analysis of other GcvB-regulated mRNAs, this approach might provide further insight into how the ACA motif participates in the translation initiation step.

## Materials and Methods

### Bacterial strains and culture conditions

Strains used in this study were derivatives of *Salmonella enterica* serovar Typhimurium strain LT2 [40]. Strain SV4280 was a gift of J. Casadesús. Except for the latter strain and for strain MA7224, all other strains were derived from MA3409, an LT2 derivative cured for the Gifsy-1 prophage [41]. The genotypes of the relevant strains used are listed in Table S1. Bacteria were cultured at 37°C in liquid media or in media solidified by the addition of 1.5% Difco agar. LB broth [42] was used as complex medium. Carbon-free medium (NCE) [43], supplemented with 0.2% glycerol or 0.2% lactose was used as minimal medium. Antibiotics (Sigma-Aldrich) were included at the following final concentrations: chloramphenicol, 10 µg ml^−1^; kanamycin monosulphate, 50 µg ml^−1^; sodium ampicillin 100 µg ml^−1^; spectinomycin dihydrochloride, 80 µg ml^−1^; tetracycline hydrochloride, 25 µg ml^−1^. MacConkey agar plates containing 1% lactose [44] were used to monitor *lacZ* expression in bacterial colonies. Liquid cultures were grown in New Brunswick gyratory shakers and growth was monitored by measuring the optical density at 600 nm with a Shimazu UV-mini 1240 spectrophotometer.

### Relevant enzymes and chemicals

T4 polynucleotide kinase and Taq DNA polymerase were from New England Biolabs, Pfu-Turbo DNA polymerase was from Stratagene, T4 DNA ligase was from New England Biolabs. DNA oligonucleotides were custom synthesized by Sigma Aldrich or Eurofins MWG/Operon. The complete list of DNA oligonucleotides used in this study is shown in Table S2. DNA sequencing was performed by GATC biotech. Acrylamide-bisacrylamide and other electrophoresis reagents were from BioRad. Agarose was from Invitrogen. Hybond-N^+^ membranes and hybridization buffer used for Northern blot analysis were from GE Healthcare and from Applied Biosystems-Ambion, respectively. The rNTPs were from Promega and the ^32^P-NTPs were from PerkinElmer or Hartmann Analytic. ^32^P-labeled nucleic acids were detected by phosphorimaging using ImageQuant software.

### Genetic techniques

Generalized transduction was performed using the high-frequency transducing mutant of phage P22, HT 105/1 *int-201* [45] as described [46]. Chromosomal engineering (recombineering) was carried out by the λ *red* recombination method [47]–[49] implemented as in [47]. Donor DNA fragments were generated by PCR using plasmid DNA or chromosomal DNA or DNA oligonucleotides as templates. Amplified fragments were electroporated into appropriate strains harboring the conditionally replicating plasmid pKD46, which carries the λ *red* operon under the control of the P^BAD^ promoter [47]. Bacteria carrying pKD46 were grown at 30°C in the presence of ampicillin and exposed to arabinose (10 mM) for 3 hours prior to preparation of electrocompetent cells. Electroporation was carried out using a Bio-Rad MicroPulser under the conditions specified by the manufacturer. Recombinant colonies were selected on LB plates containing the appropriate antibiotic. Constructs were verified by PCR and DNA sequence analysis (performed by GATC company).

### Random PCR mutagenesis

PCR amplification of DNA fragments under error-prone conditions was carried out as previously described [50].

### “Scarless” DNA recombineering

Scarless modification of chromosomal DNA sequences at the single base-pair level was achieved with a two-step recombineering procedure as previously described [51]. Briefly, this involved: 1) inserting a *tetAR* module (produced by PCR) at the chromosomal site to be modified and: 2) replacing the *tetAR* module by a DNA fragment carrying the desired changed through positive selection tetracycline-sensitive recombinants [52]. Typically, the DNA fragment in the second step was also obtained by PCR using oligonucleotides with complementary sequences at their 3′ ends priming DNA synthesis on each other (“reciprocal priming”). In site-directed mutagenesis experiments, one of the two primers contained the desired nucleotide changes or randomized sequence stretches. All constructs were verified by DNA sequencing. Table S3 shows the list of alleles made by standard or scarless recombineering.

### RNA extraction and analysis by primer extension and Northern blotting

RNA was prepared by the acid-hot-phenol method from exponentially growing cells (OD_600_ of 0.35) as previously described [50]. Reverse transcriptase reactions (enzyme Superscript II from Invitrogen) were carried out using 5 µg of bulk RNA and ^32^P-labeled primer ppF49. The same DNA primer was used for the sequencing reactions. Reactions were performed with the *fmol* DNA Cycle Sequencing System from Promega, according to the manufacturer's protocol. Reaction products were fractionated on a 10% polyacrylamide-8 M urea gel. For Northern blot analysis, RNA was fractionated on a 1% agarose-formaldehyde gel, blotted onto a nylon membrane, and hybridized to the appropriate ^32^P-labeled DNA oligonucleotide probes.

### *In vitro* translation

*In vitro* coupled transcription/translation was performed using New England Biolabs' PURExpress In vitro Protein Synthesis kit (NEB #E6800) according to the manufacturer instructions. Genes to be analyzed were cloned under T7 promoter control in the DFRH plasmid provided with the kit. The hybrid genes carried *yifK* wt or mutant 5′ UTR sequences fused to the *cat*-3×FLAG coding sequence (chloramphenicol acetyl transferase in-frame fusion to the 3×FLAG epitope). Final volume of the transcription/translation reaction was 25 µl in all cases. In addition to kit solutions A and B, reaction mix contained, 10 U of RNase inhibitor SUPERase (Ambion) and template plasmid DNA added to either 0.5 or 5 pM final concentration. Incubation times at 37°C varied from 15 to 90 min. Reactions were stopped by addition of equal volume of 2× Laemmli buffer and immediate freezing. Aliquots were loaded on 12.5% Acrylamide gels and Western analysis performed as previously described [53].

### Toeprinting assay

Toeprinting reactions were carried out as described by Darfeuille *et al* [54] with minor modifications. RNA fragments spanning positions +1 to +135 of *yifK* mRNA were synthesized *in vitro* from T7 DNA templates generated by PCR amplification of chromosomal DNA (from strains MA8020 or MA11793) with primers ppI22 and ppI23. 2 pmol of RNA were annealed with 5′end-labeled primer ppI23 (1 pmol) in 10 mM Tris-acetate [pH 7.6], 0.1 M potassium acetate, and 1 mM DTT for 1 min at 90°C and chilled in ice for 5 min. Then, all dNTPs (final concentration 1 mM), Mg Acetate (10 mM final) were added; this was followed by preincubation with 2 pmol of 30S ribosomal subunit (a gift of Dominique Fourmy and Satoko Yoshizawa) at 37°C for 5 min. In experiments involving GcvB, 5, 1 or 0.5 pmol of sRNA were added prior to both, addition of the 30S ribosomal subunit and the preincubation step. After the 5-min period, 2 pmol of tRNA^fMet^ were added and preincubation at 37°C continued for 15 additional min. Finally, Reverse Transcriptase (Superscript II, Invitrogen, 200U) was added and samples incubated for 15 min at 37°C. Following phenol chloroform extraction and ethanol precipitation, resuspended samples were loaded onto a 10% polyacrylamide-8 M urea gel along with the sequencing reaction samples generated with the same primer.

### Measurement of β-galactosidase activity

β-galactosidase activity was assayed in toluene-permeabilized cells as described in [55] and is expressed in Miller units throughout this work. Typically, measurements were performed on duplicate or triplicate cultures grown in late exponential phase (OD_600_≈0.7). All experiments included parental or reference strains as normalization controls. Standard deviations were generally less than 5% of the mean.

## Supporting Information

Figure S1 *gcvB*-linked mutations relieving *yifK* repression. A DNA fragment spanning the *gcvB* gene and a linked *cat* marker (*ygdI*::*cat*; placed 71 bp downstream in a parallel orientation) was amplified by PCR under error-prone conditions using oligonucleotides ppF17 and ppF18 as primers (Table S2) and chromosomal DNA from strain MA1179 (Table S1) as template. The amplified fragment was introduced into strain MA10280 (*yifK-lacZY*/pKD46) and recombinants were selected on MacConkey lactose plates supplemented with chloramphenicol as described in the text. Red-colored colonies were picked and the region of the *gcvB* locus analyzed by DNA sequencing. Most of the isolates were found to harbor DNA sequence changes in the *gcvA-gcvB* intergenic region, which affected either the -35 or -10 box of the *gcvB* promoter, or the -10 box of the *gcvA* promoter [18]. Mutations within the initial portion of the *gcvA* coding sequence and a mutation affecting the CG-rich stem of *gcvB*'s Rho-independent terminator were also identified (data not shown).
(TIF)

Figure S2 Comparing GcvB sRNA levels in wild-type and in an *lrp* insertion mutant as a function of the growth medium. Bacteria were grown in minimal medium (NCE [43]) supplemented with 0.2% glycerol or in LB to an OD_600_≈0.4. RNA was extracted, fractionated on an 8% polyacrylamide-8 M urea gel and subjected to Northern blot hybridization. Blot was hybridized to DNA oligonucleotides complementary to GcvB and to 5S RNA (for loading control). The probes used were ppI67 (GcvB) and ppB10 (5S)(Table S2).
(TIF)

Figure S3 Regulation of *dppA-lacZ* fusion by GcvB variants. GcvB alleles GA*^84,85^* and ACA*^86–88^* fall outside the portion of GcvB that pairs with *dppA* [16]. The two mutants are as effective as GcvB*^WT^* in down-regulating *dppA-lacZ*. This shows that the sequence changes do not affect the functioning of the GcvB. The Δ symbol denotes a complete deletion of the *gcvB* gene.
(TIF)

Figure S4 Alignment of sequences preceding *yifK* translation start site in members of the *Enterobacteriaceae* family. Sequences (from NCBI database) are 100% identical among isolates from the same species. Green shading denotes conservation of the ACA motifs analyzed in in this study.
(TIF)

Figure S5 A *yifK* variant with optimal spacing between the SD and the initiator AUG still depends on the upstream ACA for optimal expression. Replacing the upstream ACA with GGG lowers expression as well as the response to GcvB repression (see main text and Figure 9 for details).
(TIF)

Table S1 Relevant strains used in this work. Strains are derived from *Salmonella enterica* serovar Typhimurium strain MA3409 [41] except for strains SV4280 [56], MA3398 [41] and MA7224 [53]. The term “scar” denotes the DNA sequence left following Flp-mediated excision of antibiotic-resistance cassettes introduced by the procedure of Datsenko and Wanner [47]. The *dppA*::pCE40 (*lacZY*) fusion was constructed as described [57]. Additional details on relevant alleles in this table can be found in Table S3.
(DOCX)

Table S2 DNA oligonucleotides used in this work. Changes from the wild-type sequence are in red. Nucleotide insertions are in bold and underlined. “N” denotes an equimolar mixture of all four nucleotides, “V” denotes an equimolar mixture of A, C and G; “H” denotes an equimolar mixture of A, T and C.
(DOCX)

Table S3 Relevant alleles constructed in this work. Template DNA was from plasmids (pSEB5 [21] and pKD3 [47]) or chromosome (“MA” numbers identify the *Salmonella* strains used as source of DNA; described in the text) or from primers annealing to each other (“self”). Further details on DNA oligonucleotide primers are given in Table S2.
(DOCX)
