# Supplementary figures and images for: Translation Enhancing ACA Motifs and Their Silencing by a Bacterial Small Regulatory RNA
Source: PLoS Genet. 2014 Jan 2;10(1):e1004026. doi: 10.1371/journal.pgen.1004026 (PMC3879156; doi:10.1371/journal.pgen.1004026)

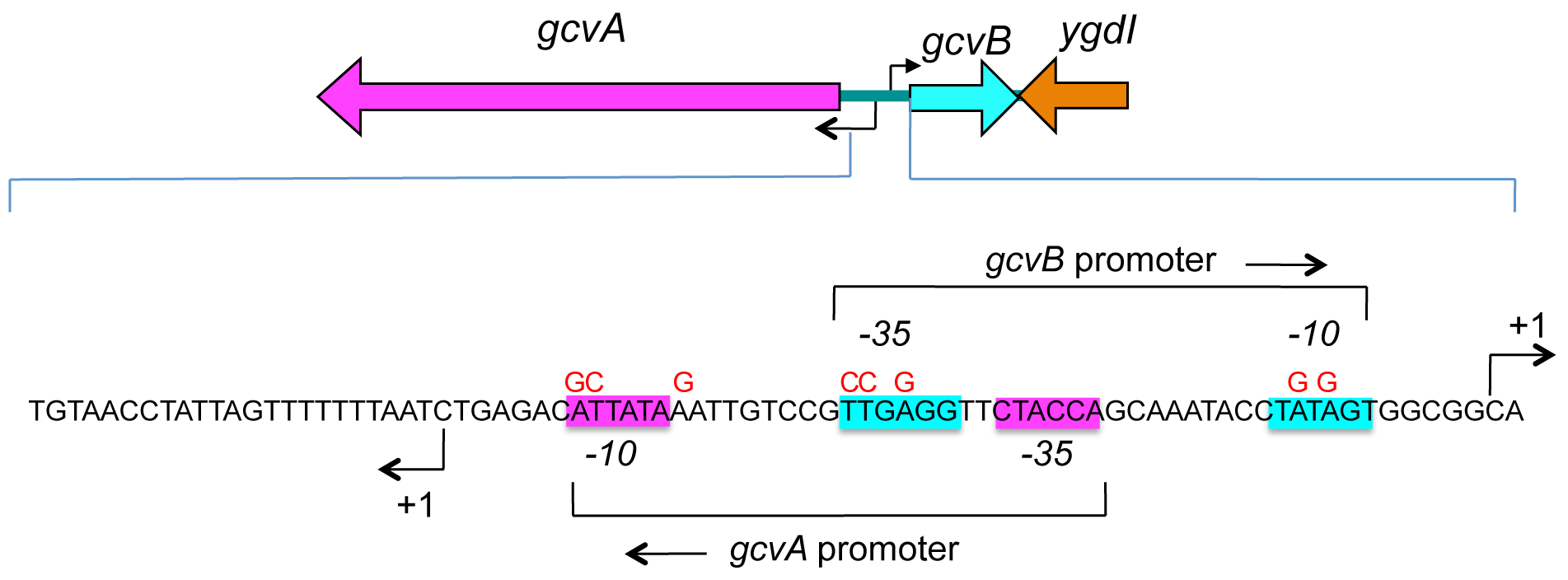

Supplement: Figure S1 — gcvB-linked mutations relieving yifK repression. A DNA fragment spanning the gcvB gene and a linked cat marker (ygdI::cat; placed 71 bp downstream in a parallel orientation) was amplified by PCR under error-prone conditions using oligonucleotides ppF17 and ppF18 as primers (Table S2) and chromosomal DNA from strain MA1179 (Table S1) as template. The amplified fragment was introduced into strain MA10280 (yifK-lacZY/pKD46) and recombinants were selected on MacConkey lactose plates supplemented with chloramphenicol as described in the text. Red-colored colonies were picked and the region of the gcvB locus analyzed by DNA sequencing. Most of the isolates were found to harbor DNA sequence changes in the gcvA-gcvB intergenic region, which affected either the -35 or -10 box of the gcvB promoter, or the -10 box of the gcvA promoter [18]. Mutations within the initial portion of the gcvA coding sequence and a mutation affecting the CG-rich stem of gcvB's Rho-independent terminator were also identified (data not shown). (TIF) [file pgen.1004026.s001.tif]

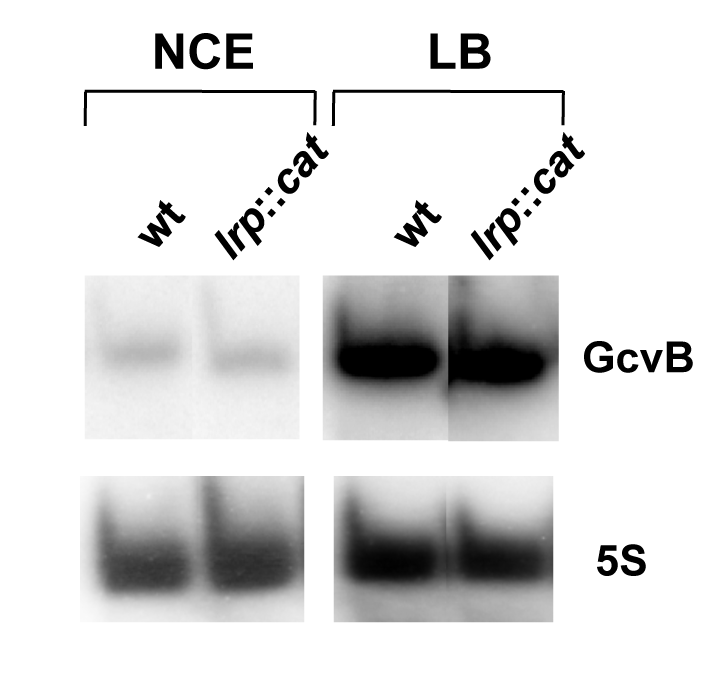

Supplement: Figure S2 — Comparing GcvB sRNA levels in wild-type and in an lrp insertion mutant as a function of the growth medium. Bacteria were grown in minimal medium (NCE [43]) supplemented with 0.2% glycerol or in LB to an OD600≈0.4. RNA was extracted, fractionated on an 8% polyacrylamide-8 M urea gel and subjected to Northern blot hybridization. Blot was hybridized to DNA oligonucleotides complementary to GcvB and to 5S RNA (for loading control). The probes used were ppI67 (GcvB) and ppB10 (5S)(Table S2). (TIF) [file pgen.1004026.s002.tif]

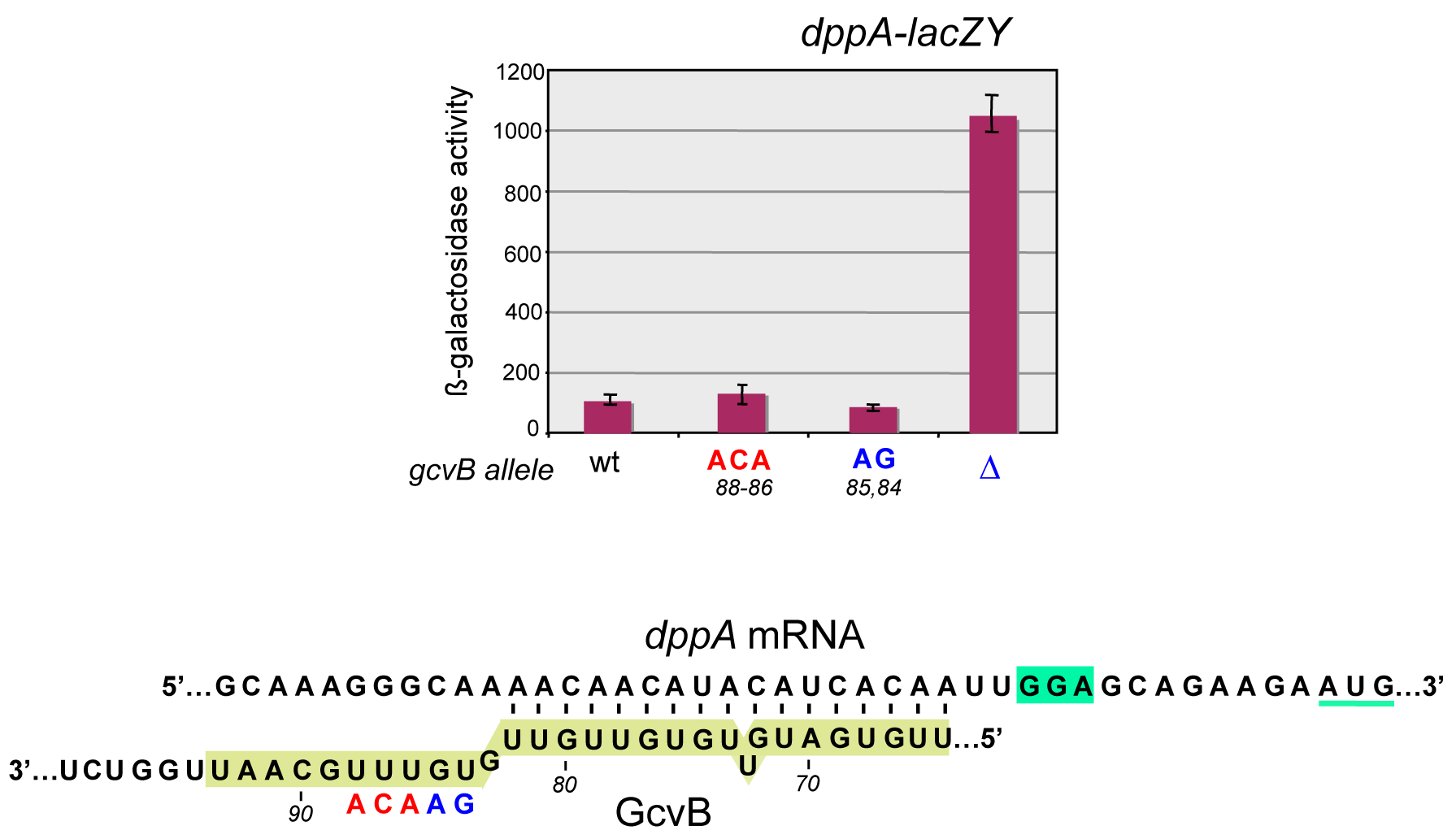

Supplement: Figure S3 — Regulation of dppA-lacZ fusion by GcvB variants. GcvB alleles GA84,85 and ACA86–88 fall outside the portion of GcvB that pairs with dppA [16]. The two mutants are as effective as GcvBWT in down-regulating dppA-lacZ. This shows that the sequence changes do not affect the functioning of the GcvB. The Δ symbol denotes a complete deletion of the gcvB gene. (TIF) [file pgen.1004026.s003.tif]

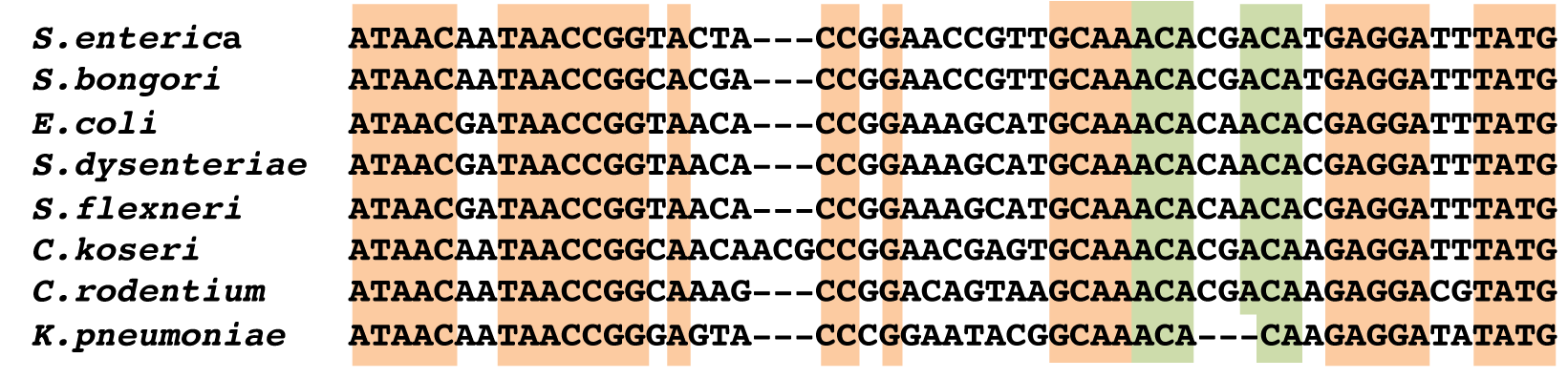

Supplement: Figure S4 — Alignment of sequences preceding yifK translation start site in members of the Enterobacteriaceae family. Sequences (from NCBI database) are 100% identical among isolates from the same species. Green shading denotes conservation of the ACA motifs analyzed in in this study. (TIF) [file pgen.1004026.s004.tif]

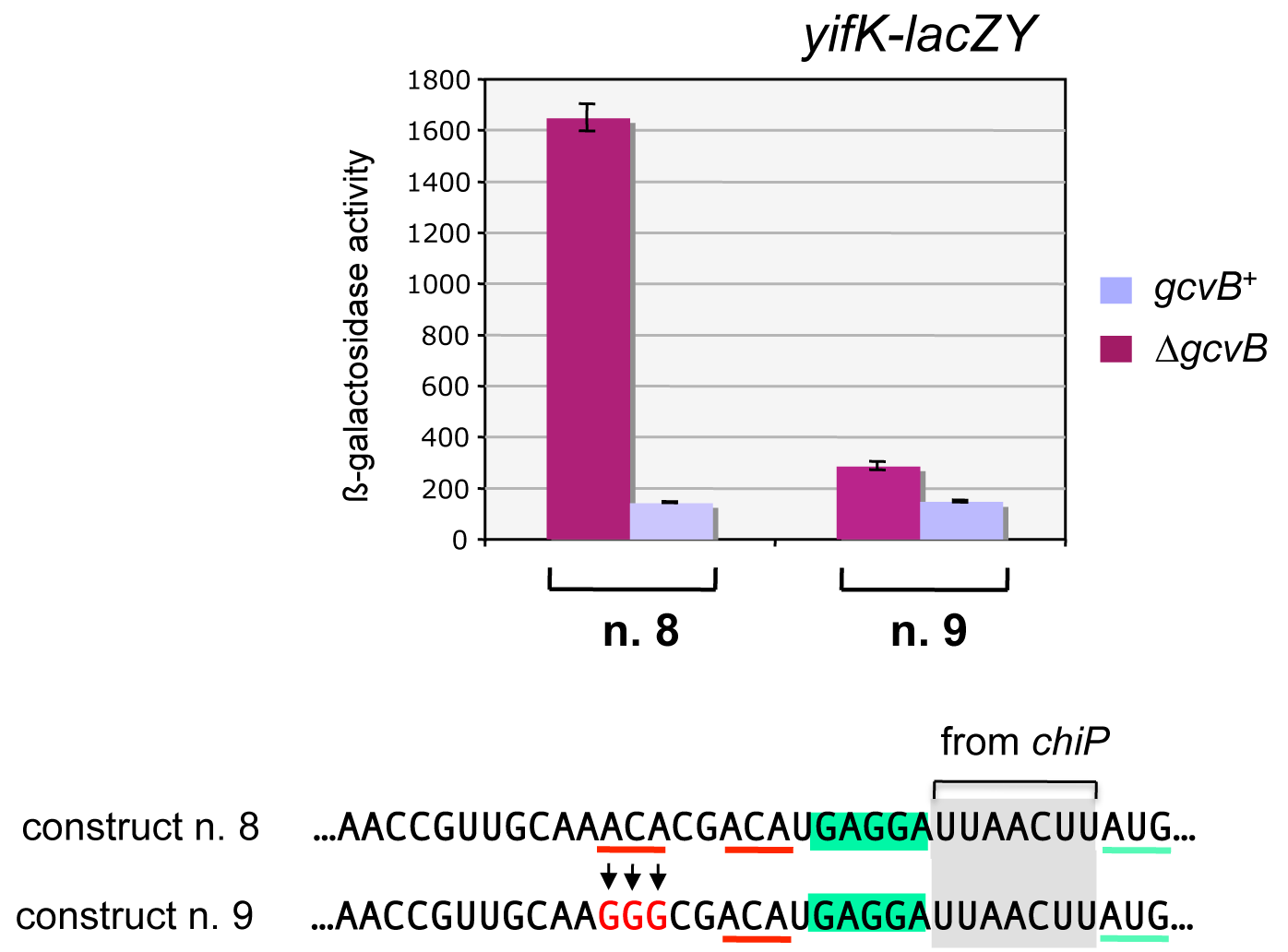

Supplement: Figure S5 — A yifK variant with optimal spacing between the SD and the initiator AUG still depends on the upstream ACA for optimal expression. Replacing the upstream ACA with GGG lowers expression as well as the response to GcvB repression (see main text and Figure 9 for details). (TIF) [file pgen.1004026.s005.tif]
