# Supplementary material for: Translation Enhancing ACA Motifs and Their Silencing by a Bacterial Small Regulatory RNA
Source: PLoS Genet. 2014 Jan 2;10(1):e1004026. doi: 10.1371/journal.pgen.1004026 (PMC3879156; doi:10.1371/journal.pgen.1004026)
Supplement: Table S1 — Relevant strains used in this work. Strains are derived from Salmonella enterica serovar Typhimurium strain MA3409 [41] except for strains SV4280 [56], MA3398 [41] and MA7224 [53]. The term “scar” denotes the DNA sequence left following Flp-mediated excision of antibiotic-resistance cassettes introduced by the procedure of Datsenko and Wanner [47]. The dppA::pCE40 (lacZY) fusion was constructed as described [57]. Additional details on relevant alleles in this table can be found in Table S3. (DOCX) [file pgen.1004026.s006.docx]

| Strain^a^ | Genotype | Source or reference |
| --- | --- | --- |
| MA3398 | *zfh-8157*::Tn*10*dTc | [41] |
| MA3409 | wild-type | [41] |
| MA7224 | *sodCI*::3xFLAG ilvI3305::Tn*10*dTac-*cat*-3xFLAG | [53] |
| MA7455 | wild-type / pKD46 | [21] |
| MA8020 | *yifK87*::MudK | [21] |
| MA8021 | *yifK87*::MudK *∆hfq67*::*cat* | [21] |
| MA8201 | *eptB115*::*MudK hfq13*::Tn*5*-T-POP (TcR) | [21] |
| MA9791 | [*rluC-rne*]IG::*cat rne-3071* (ts) | [28] |
| SV4280 | *lrp-42*::Tn*10*dCm | [56] |
| MA10241 | ∆*gcvB141*::*cat* | this work |
| MA10242 | ∆*gcvB142*:: *aadA* (Spec^R^) | this work |
| MA10280 | *yifK87*::MudK / pKD46 | this work |
| MA10377 | *yifK87*::MudK ∆*gcvB141*::*cat* | this work |
| MA10403 | *yifK87*::MudK ∆*gcvB141*::*cat* *∆hfq67*::*cat* | this work |
| MA11526 | *yifK145*::*tetAR* / pKD46 | this work |
| MA11559 | *yifK145*::*tetAR yifK87*::MudK / pKD46 | this work |
| MA11594 | *yifK145*::*tetAR yifK87*::MudK ∆*gcvB141*::*cat* / pKD46 | this work |
| MA11779 | *ygdI143*::*cat* | this work |
| MA11780 | *wecG144*::*cat* | this work |
| MA11781 | *yifK*[-33A] *yifK87*::MudK *wecG144*::*cat* | this work |
| MA11782 | *yifK*[21C] *yifK87*::MudK *wecG144*::*cat* | this work |
| MA11783 | *yifK*[27A] *yifK87*::MudK *wecG144*::*cat* | this work |
| MA11784 | *yifK*[-33A] *yifK87*::MudK | this work |
| MA11785 | *yifK*[-33A] | this work |
| MA11786 | *yifK*[21C] *yifK87*::MudK *wecG144*::*cat* ∆*gcvB141*::scar | this work |
| MA11787 | *yifK*[27A] *yifK87*::MudK *wecG144*::*cat* ∆*gcvB141*::scar | this work |
| MA11788 | *yifK*[21C] *yifK87*::MudK *wecG144*::*cat* *hfq13::*Tn*5*-T-POP | this work |

| Strain^a^ | Genotype | Source or reference |
| --- | --- | --- |
| MA11789 | *yifK*[27A] *yifK87*::MudK *wecG144*::*cat* *hfq13*::Tn*5*-T-POP | this work |
| MA11790 | *yifK*[46-48UGU] *yifK87*::MudK | this work |
| MA11791 | *yifK87*::MudK *gcvB*[86-88ACA] *ygdI143*::*cat* | this work |
| MA11792 | *yifK*[46-48UGU] *yifK87*::MudK *gcvB*[86-88ACA] *ygdI143*::*cat* | this work |
| MA11793 | *yifK*[49,50UC] *yifK87*::MudK | this work |
| MA11794 | *yifK87*::MudK *gcvB*[84,85GA] *ygdI143*::*cat* | this work |
| MA11795 | *yifK*[49,50UC] *yifK87*::MudK *gcvB*[84,85GA] *ygdI143*::*cat* | this work |
| MA11796 | *yifK*[49,50UC] *yifK87*::MudK ∆*gcvB141*::*cat* | this work |
| MA11797 | *yifK*[49,50UC] *yifK87*::MudK ∆*hfq67*::*cat* | this work |
| MA11798 | *yifK*[-33A] ∆*yifK146*::*cat-*3xFLAG | this work |
| MA11799 | *yifK*[-33A] *yifK*[49,50UC] *∆yifK146::cat-*3xFLAG | this work |
| MA11800 | ∆*gcvB141*::scar [*rluC-rne*] IG::*cat rne-3071* (ts) | this work |
| MA11801 | *yifK*[-33A] ∆*gcvB141*::*cat* | this work |
| MA11802 | *yifK*[-33A] [*rluC-rne*]IG::*cat rne-3071* (ts) | this work |
| MA11803 | *yifK*[-33A] *hfq13::*Tn*5*-T-POP | this work |
| MA11804 | *yifK*[-33A] ∆*gcvB142*::*aadA* [*rluC-rne*]IG::*cat rne-3071* (ts) | this work |
| MA11805 | *yifK*[-33A] ∆*gcvB141*::*cat hfq13::*Tn*5*-T-POP | this work |
| MA11806 | *yifK87*::MudK *lrp-42*::Tn*10*dCm | this work |
| MA11807 | *yifK87*::MudK ∆*gcvB142*::*aadA* | this work |
| MA11808 | *yifK87*::MudK ∆*gcvB142*::*aadA lrp-42*::Tn*10*dCm | this work |
| MA11847 | *dppA147*::pCE40 (*lac*) ∆*gcvB141*::*cat* / pKD46 | this work |
| MA11848 | *dppA149*::*tetAR dppA147*::pCE40 (*lac*) ∆*gcvB141*::*cat* / pKD46 | this work |
| MA11849 | *yifK*[-33A] *yifK*[G59C] | this work |
| MA11855 | *yifK151* *yifK87*::MudK ∆*gcvB141*::*cat* | this work |
| MA11856 | *yifK152* (ACA > GGG) *yifK87*::MudK ∆*gcvB141*::*cat* | this work |
| MA11863 | *yifK151* *yifK87*::MudK | this work |
| MA11864 | *yifK152* (ACA > GGG) *yifK87*::MudK | this work |

| Strain^a^ | Genotype | Source or reference |
| --- | --- | --- |
| MA11867 | *yifK*[-33A] ∆*gcvB141*::*cat* | this work |
| MA11868 | *yifK*[-33A] *∆hfq67*::*cat* | this work |
| MA11869 | *yifK*[-33A] [*rluC-rne*]IG::*cat rne-3071* (ts) | this work |
| MA11870 | *yifK*[-33A] *yifK*[G59C] ∆*gcvB141*::*cat* | this work |
| MA11871 | *yifK*[-33A] *yifK*[G59C] *∆hfq67*::*cat* | this work |
| MA11872 | *yifK*[-33A] *yifK*[G59C] [*rluC-rne*]IG::*cat rne-3071* (ts) | this work |
| MA11875 | *yifK87*::MudK *lrp-42*::Tn*10*dCm | this work |
| MA11876 | *yifK87*::MudK ∆*gcvB142*::*aadA* | this work |
| MA11877 | ∆*gcvB142*::*aadA lrp-42*::Tn*10*dCm | this work |
