# Supplementary material for: Translation Enhancing ACA Motifs and Their Silencing by a Bacterial Small Regulatory RNA
Source: PLoS Genet. 2014 Jan 2;10(1):e1004026. doi: 10.1371/journal.pgen.1004026 (PMC3879156; doi:10.1371/journal.pgen.1004026)
Supplement: Table S2 — DNA oligonucleotides used in this work. Changes from the wild-type sequence are in red. Nucleotide insertions are in bold and underlined. “N” denotes an equimolar mixture of all four nucleotides, “V” denotes an equimolar mixture of A, C and G; “H” denotes an equimolar mixture of A, T and C. (DOCX) [file pgen.1004026.s007.docx]

| name | Sequence (5'-3')^a^ |
| --- | --- |
| pp813 | GCGGAGGCTAGGGAGAGAGG |
| ppB10 | ACACTACCATCGGCGCTACG |
| ppF08 | ATTGTCCGTTGAGGTTCTACCAGCAAATACCTATAGTGGCCCATGGTCCATATGAATATC |
| ppF09 | TACTGACGTGAAAGAGATGGTGGCTCTGGAAAACTAACCGTGTGTAGGCTGGAGCTGCTT |
| ppF10 | ATTGTCCGTTGAGGTTCTACCAGCAAATACCTATAGTGGCACCTTGCCGTAGAAGAACAG |
| ppF11 | TACTGACGTGAAAGAGATGGTGGCTCTGGAAAACTAACCGTTTGGCTGTGAGCAATTATG |
| ppF16 | GGCAATCAATTCAATATGACGAGCTTCCAGCCCACGC |
| ppF17 | CATCTCTTTCACGTCAGTACGATTGATCTGCTGTTTGTTGCCATGGTCCATATGAATATC |
| ppF18 | GTCCTAATTACGTTATGCACACCAATGATGGACGTAGCATTGTGTAGGCTGGAGCTGCTT |
| ppF19 | TGCTCGCGTAAAGCTCAAAT |
| ppF20 | CGCAAACTGGCTACGATGAA |
| ppF43 | TATCTTCGCTGGCACTATACTGGCGATCTCTAATTCTCCCTGTGTAGGCTGGAGCTGCTT |
| ppF44 | CATCGATTATGTTGTAAATGAGCAGCACCATAAGCACAATCCATGGTCCATATGAATATC |
| ppF45 | CGTGGTGCGTTCAATACGTA |
| ppF46 | CGCCATCCACATAAACCAGT |
| ppF49 | TCTCTGCCATAAATCCTCAT |
| ppF62 | CGCCCATTTCAGCGTACT |
| ppG42 | AGGGCAGCGCTCTATCCAGCTGAGCTACGGGCGCTTAG |
| ppG44 | AATCGATGTACCCATAACAATAACCGGTACTACCGGAACCTTAAGACCCACTTTCACATT |
| ppG45 | ACGCTGTAGCTCCGGTTTTTTCTCTGCCATAAATCCTCATCTAAGCACTTGTCTCCTG |
| ppG48 | TACCCATAACAATAACCGGTACTACCGGAACCGTTGC**NNN**CACGACATGAGGATTTATGGCAG |
| ppG49 | CGAGCTTCCAGCCCACGCTGTAGCTCCGGTTTTTTCTCTGCCATAAATCCTCATGTCGTG |
| ppG51 | TATGACGAGCTTCCAGCCCACGCTGTAGCTCCGGTTTTTTCTCTGCCATAAATCCTCATG |
| ppG63 | TTCTGATGGGCTTTTGGCTTACGGTTGTGATGTTGTGTTGTTGTG**ACA**GCAATTGGTCTGCGATT |
| ppH12 | TCGATGTACCCATAACAATAACCGGTACTACCGGAACCGTTGCAAA**NN**CGACATGAGGATTTATGGCAG |
| ppH13 | TATGACGAGCTTCCAGCCCACGCTGTAGCTCCGGTTTTTTCTCTGCCATAAATCCTCATGTCG |
| ppH27 | TGAAACTTTTTACGCGGTATTAAACCACCGCAGCTCAAGCACCTAAATAAA |
| ppH61 | CTGATGGGCTTTTGGCTTACGGTTGTGATGTTGTGTTGTTG**GA**TTTGCAATTGGTCTGCGATTC |
| ppH81 | CGATGATTTCTTACACAATAAGTGCATTTTTTTAATGCTCCATTTG**A**CATTTGTCCAAATTTAAG |
| ppI22 | GGTAATACGACTCACTATAGGGCAATCGATGTACCCATAACAATAACCG |
| ppI23 | CCCGAGGGCAATCAATTCAA |
| ppI67 | AGACCAATTGCAAACACAACAACACAACATCACAACCGTAAGCCA |
| ppL50 | TACTACCGGAACCGTTGCAAACACGACATGAGGATTTATGGAGAAAAAAATCACTGGATATACCA |
| ppL51 | TACTACCGGAACCGTTGCAAA**TC**CGACATGAGGATTTATGGAGAAAAAAATCACTGGATATACC |
| ppL52 | AAATGCGCGTTTGGTTATGCTTTGCGCATTTTGGCGCTTACTATTTATCGTCGTCATCTTTGTAG |
| ppL95 | AATCGATGTACCCATAACAATAACCGGTACTACCGGAACCGTTGCAA |
| ppL96 | ACGCTGTAGCTCCGGTTTTTTCTCTGCCATAAATCCTCATGTCG**NNN**TTGCAACGGTTCCGGTAGTAC |

| name | Sequence (5'-3')^a^ |
| --- | --- |
| ppL97 | AATCGATGTACCCATAACAATAACCGGTACTACCGGAACCGTTGCAAACACG |
| ppL98 | CACGCTGTAGCTCCGGTTTTTTCTCTGCCATAAATCCTCA**NNN**CGTGTTTGCAACGGTTCCGGT |
| ppM29 | AATTTATATTTTAACTATTCTAGACAATCGATGTACCCATAACAATAACC |
| ppM30 | ATTTATATTTCGGCCGCTCGAGCTGCAGTGGCGCTTACTATTTATCGTCG |
| ppM80 | ACGCTGTAGCTCCGGTTTTTTCTCTGCCATAAATCCTCATGTCGTG**V**TTGCAACGGTTCCGGTAGTAC |
| ppM81 | ACGCTGTAGCTCCGGTTTTTTCTCTGCCATAAATCCTCATGTCGT**H**TTTGCAACGGTTCCGGTAGTAC |
| ppM82 | ACGCTGTAGCTCCGGTTTTTTCTCTGCCATAAATCCTCATGTCG**V**GTTTGCAACGGTTCCGGTAGTAC |
| ppM83 | CACGCTGTAGCTCCGGTTTTTTCTCTGCCATAAATCCTCATG**V**CGTGTTTGCAACGGTTCCGGT |
| ppM84 | CACGCTGTAGCTCCGGTTTTTTCTCTGCCATAAATCCTCAT**H**TCGTGTTTGCAACGGTTCCGGT |
| ppM85 | CACGCTGTAGCTCCGGTTTTTTCTCTGCCATAAATCCTCA**V**GTCGTGTTTGCAACGGTTCCGGT |
| ppN58 | CAGCCCACGCTGTAGCTCCGGTTTTTTCTCTGCCATAAATCCTCATG**CCTCATG**TCGTGTTTGCAACGGTTCCG |
| ppN59 | CAGCCCACGCTGTAGCTCCGGTTTTTTCTCTGCCATAAATC**G**TCATG**CCTCATG**TCGTGTTTGCAACGGTTCCG |
| ppN60 | AATCGATGTACCCATAACAATAACCGGTACTACCGGAACCGTTGCAAACACGACA |
| ppN63 | CAGCCCACGCTGTAGCTCCGGTTTTTTCTCTGCCATAAATC**G**TCATGTCGTGTTTGCAACGGTTCCG |
| ppN64 | CAGCCCACGCTGTAGCTCCGGTTTTTTCTCTGCCATAAATCCTCATG**CGTCATG**TCGTGTTTGCAACGGTTCCG |
| ppN73 | AATCGATGTACCCATAACAATAACCGGTACTACCGGAACCGTTGCAA**GGG**CGACATG |
| ppN74 | CAGCCCACGCTGTAGCTCCGGTTTTTTCTCTGCCATAAATCCTCATG**CGTCATG**TCG**CCC**TTGCAACGGTTC |
| ppN75 | CAGCCCACGCTGTAGCTCCGGTTTTTTCTCTGCCATAAATC**G**TCATG**CCTCATG**TCG**CCC**TTGCAACGGTTC |
| ppN76 | TGTACCCATAACAATAACCGGTACTACCGGAACCGTTGCAA**NNN**CGA**CATGACG**CATGAGGATTTATGG |
| ppN77 | CAGCCCACGCTGTAGCTCCGGTTTTTTCTCTGCCATAAATCCTCATGC**G**TCATGTCG |
| ppN78 | TGTACCCATAACAATAACCGGTACTACCGGAACCGTTGCAA**NNN**CGA**CATGAGG**CATGA**C**GATTTATGG |
| ppN79 | CAGCCCACGCTGTAGCTCCGGTTTTTTCTCTGCCATAAATC**G**TCATGCCTCATGTCG |
| ppN82 | ACAAAAAAGGCCGGGCGGTAAAAGCCTTTGCAAAGGGCAAATTAAGACCCACTTTCACATT |
| ppN83 | ATCCCTGACTTCTTCAAGGAAATACTCATTCTTCTGCTCCAATCTAAGCACTTGTCTCCTG |
| ppN84 | AACAAAAAAGGCCGGGCGGTAAAAGCCTTTGCAAAGGGCAAAATTGGAGCAGAAGAATGAGTATTTCC |
| ppN85 | AAAAAGGCCGGGCGGTAAAAGCCTTTGCAAAGGGCAAA**NNNNNN**T**NNN**TC**NNN**ATTGGAGCAGAAGAATGAGT  ATTTCC |
| ppN86 | ATCCCTGACTTCTTCAAGGAAATACTCATTCTTCTGCTCCAAT |
| ppO1 | AATCGATGTACCCATAACAATAACCGGTACTACCGGAACCGTTGCAAACACGACATGAGG |
| ppO2 | CTTCCAGCCCACGCTGTAGCTCCGGTTTTTTCTCTGCCATAAGTTAATCCTCATGTCGTGTTTGCAACGG |
| ppO3 | AATCGATGTACCCATAACAATAACCGGTACTACCGGAACCGTTGCAA**GGG**CGACATGAGG |
| ppO4 | CTTCCAGCCCACGCTGTAGCTCCGGTTTTTTCTCTGCCATAAGTTAATCCTCATGTCGCCCTTGCAACGG |
