# Supplementary material for: Translation Enhancing ACA Motifs and Their Silencing by a Bacterial Small Regulatory RNA
Source: PLoS Genet. 2014 Jan 2;10(1):e1004026. doi: 10.1371/journal.pgen.1004026 (PMC3879156; doi:10.1371/journal.pgen.1004026)
Supplement: Table S3 — Relevant alleles constructed in this work. Template DNA was from plasmids (pSEB5 [21] and pKD3 [47]) or chromosome (“MA” numbers identify the Salmonella strains used as source of DNA; described in the text) or from primers annealing to each other (“self”). Further details on DNA oligonucleotide primers are given in Table S2. (DOCX) [file pgen.1004026.s008.docx]

| Primer pair | Template^a^ | Allele | Description |
| --- | --- | --- | --- |
| ppF08 / ppF09 | pKD3 | ∆*gcvB141*::c*at* | *cat* gene in place of *gcvB* |
| ppF10 / ppF12 | pSEB5 | ∆*gcvB142*::*aadA* | Spec^R^ gene in place of *gcvB* |
| ppF17 / ppF18 | pKD3 | *ygdI143*::*cat* | *cat* on 3’ side of *gcvB* (parallel orientation) |
| ppF43 / ppF44 | pKD3 | *wecG144*::*cat* | *cat* on 5’ side of *yifK* (opposite orientation) |
| ppG44 / ppG45 | MA3397 | *yifK145*::*tetAR* | *tetAR* insertion 10 bp upstream of *yifK* AUG |
| ppG48 / ppG49 | self | *yifK*[46-48 UGU] | Replacement of *yifK* mRNA segment between +46 and +48 (AAA to UGU) |
| ppG63 / ppF18 | MA11779 | *gcvB*[86-88 ACA] | Replacement of GcvB segment between +86 and +88 (UUU to ACA) |
| ppH12/ ppH13 | self | *yifK*[49-50 UC] | Replacement of *yifK* mRNA segment between +49 and +50 (CA to UC) |
| ppH61 / ppF18 | MA11779 | *gcvB*[84-85 GA] | Replacement of GcvB segment between +84 and +85 (UG to GA) |
| ppH81 / ppG51 | MA3409 | *yifK*[-33A] | *yifK* promoter “up” mutation (G to A at -33) in wild-type background |
| ppL50 / ppL52 | MA7224 | *yifK*::*cat-*3xFLAG | Replacement of *yifK* orf by *cat-*3xFLAG orf |
| ppL51 / ppL52 | MA7224 | *yifK*[49-50 UC]::*cat*-3xFLAG | Replacement of *yifK* orf by *cat-*3xFLAG orf in the C49U,A50C mutant |
| ppL95 / ppL96 | self | *yifK*[48-50 NNN] | Randomized sequence in 48-50 segment of *yifK* mRNA (AUG-distal ACA] |
| ppL97 / ppL98 | self | *yifK*[53-55 NNN] | Randomized sequence in 53-55 segment of *yifK* mRNA (AUG-proximal ACA) |
| ppL95 / ppM80 | self | *yifK*[48 V] | C, U, or G at position 48 of *yifK* mRNA |
| ppL95 / ppM81 | self | *yifK*[49 H] | A, U, or G at position 49 of *yifK* mRNA |
| ppL95 / ppM82 | self | *yifK*[50 V] | C, U, or G at position 50 of *yifK* mRNA |

| Primer pair | Template^a^ | Allele | Description |
| --- | --- | --- | --- |
| ppL97 / ppM83 | self | *yifK*[53 V] | C, U, or G at position 53 of *yifK* mRNA |
| ppL97 / ppM84 | self | *yifK*[54 H] | A, U, or G at position 54 of *yifK* mRNA |
| ppL97 / ppM85 | self | *yifK*[55 V] | C, U, or G at position 55 of *yifK* mRNA |
| ppN60 / ppN58 | self | *yifK*[54-60]**_2_** | Tandem direct duplication of 54-60 segment in yifK mRNA |
| ppN60 / ppN59 | self | *yifK*[54-60]**_2_** G66C | G to C at 66 in duplicated SD background (inactivates AUG-proximal SD) |
| ppN60 / ppN63 | self | yifK[G59C] | G to C at position 59 (inactivates SD) |
| ppN60 / ppN64 | self | *yifK*[54-60]**_2_** G59C | G to C at 59 in duplicated SD background (inactivates AUG-distal SD) |
| ppN73 / ppN74 | self | *yifK*[48-50 GGG] [54-60]**_2_** G59C | ACA48-50 to GGG in duplicated background and AUG-distal SD mutated |
| ppN73 / ppN75 | self | *yifK*[48-50 GGG] [54-60]**_2_** G66C | ACA48-50 to GGG in duplicated bckgrnd and AUG-proximal SD mutated |
| ppN76 / ppN77 | self | *yifK*[48-50 NNN] [54-60]**_2_** G59C | ACA48-50 to NNN in duplicated background and AUG-distal SD mutated |
| ppN78 / ppN79 | self | *yifK*[48-50 NNN] [54-60]**_2_** G66C | ACA48-50 to NNN in duplicated bckgrnd and AUG-proximal SD mutated |
| ppN82 / ppN83 | MA3397 | *dppA149*::*tetAR* | *tetAR* insertion in *dppA* 5’ UTR; deletes four ACA repeats |
| ppN84 / ppN86 | self | *dppA150* | Deletion of 15-nt segment including four ACA repeats in *dppA* 5’ UTR |
| ppO1 / ppO2 | self | *yifK151* | Sequence between SD and AUG replaced by corresponding *chiP* region |
| ppO3 / ppO4 | self | *yifK152* | Same as *yifK151*, but upstream ACA changed to GGG |
